# Effects of 12-week 24-form Tai Chi on cognitive function, cerebral perfusion, and vascular function in postmenopausal women with mild cognitive impairment: an exploratory randomized controlled trial

**DOI:** 10.3389/fnagi.2026.1880915

**Published:** 2026-09-25

**Authors:** Yan Zheng, Lifen Hong, Lijuan Wu, Xinbei Chen, Qiaoling Hu, Liming Chen, Xuling Ni, Xiaojin Zhuang, Lianhua Yin

**Affiliations:** 1 Health Management Center, The Second Affiliated Hospital of Fujian University of Traditional Chinese Medicine, Fuzhou, Fujian, China; 2 Department of General Practice, Affiliated People's Hospital of Fujian University of Traditional Chinese Medicine, Fuzhou, Fujian, China

**Keywords:** cerebral blood flow, cerebrovascular function, cognitive function, mild cognitive impairment, nitric oxide, postmenopausal women, randomized controlled trial, Tai Chi

## Abstract

**Background:**

Postmenopausal women are at increased risk of mild cognitive impairment (MCI). Vascular and cerebrovascular dysfunction may contribute to this increased vulnerability. Tai Chi has been shown to benefit cognitive function, but its effects on cerebrovascular health in this high-risk population remain poorly defined. This study aimed to evaluate the impact of a 12-week Tai Chi intervention on cognitive performance, cerebral perfusion, and circulating vasomotor and Alzheimer’s disease (AD)-related biomarkers in postmenopausal women with MCI.

**Methods:**

This single-center, parallel-group, open-label exploratory randomized controlled trial was conducted from June 2023 to September 2024 in Fuzhou, China. Forty postmenopausal women with MCI were randomized in a 1:1 ratio using block randomization to either a 12-week supervised 24-form Tai Chi program or a health education control group. Primary and secondary outcomes included Montreal Cognitive Assessment (MoCA) total and domain-specific scores, cerebral blood flow in cognition-related brain regions, circulating vasomotor factors, and serum AD-related biomarkers.

**Results:**

Compared with the health education control group, the Tai Chi group showed greater improvement in MoCA total score (*β* = 4.95, *p* < 0.001) and in delayed recall (*β* = 1.50), visuospatial/executive function (*β* = 1.10), and attention (*β* = 1.05) (all *p* < 0.001). Tai Chi was also associated with greater increases in global and regional CBF, including a significant group-by-time interaction for global CBF (*β* = 15.29, *p* < 0.001), as well as increased plasma NO (*β* = 10.41, *p* < 0.001), a lower ET-1/NO ratio (*β* = −0.97, *p* = 0.004), and greater reductions in circulating T-tau (*β* = −255.45, *p* < 0.001), P-tau (*β* = −135.67, *p* < 0.001), and Aβ_1–42_ (*β* = −169.41, *p* < 0.001). No intervention-related adverse events occurred.

**Conclusion:**

A 12-week program of standardized 24-form Tai Chi was associated with improvements in overall cognitive performance as assessed by the MoCA in postmenopausal women with MCI. These cognitive changes were accompanied by increased cerebral perfusion, a more favorable vasomotor profile, and reductions in circulating AD-related biomarkers. Tai Chi appears to be a feasible and well-tolerated non-pharmacological intervention for this vulnerable population.

**Clinical trial registration:**

ChiCTR2300068871.

## 1 Introduction

Alzheimer’s disease (AD) is the leading cause of dementia worldwide and poses a substantial and growing socioeconomic burden. Mild cognitive impairment (MCI), widely regarded as a prodromal stage of AD, represents a critical window for early intervention, as timely management may delay or prevent progression to irreversible dementia (Mian et al., 2024). In China, MCI affects an estimated 38 million adults aged ≥ 60 years (prevalence 15.5%), with women exhibiting a 1.51-fold higher risk than age-matched men (Jia et al., 2020). Longitudinal studies indicate that approximately 25.5% of individuals with MCI progress to AD within 5 years (Clarke et al., 2022). Notably, postmenopausal women account for a disproportionate share of this burden, showing a faster rate of cognitive decline and a higher risk of conversion to AD than men (Scheyer et al., 2018).

This increased vulnerability may be partially related to the decline in endogenous estrogen following menopause (Sochocka et al., 2023). Estrogen plays a vital role in maintaining cerebrovascular homeostasis and the functional integrity of the neurovascular unit, which contributes to the regulation of cerebral blood flow (CBF) in response to neuronal metabolic demands (Owens et al., 2024; Mervosh and Devi, 2025). Disruption of these vascular regulatory mechanisms is now recognized as an early, upstream driver of AD pathogenesis (Mok et al., 2024; Mansour et al., 2025). Moreover, resting CBF provides a measure of cerebral perfusion, whereas circulating vascular biomarkers provide complementary information on systemic vascular and vasomotor function. Together, these measures may provide complementary insights into cerebrovascular health.

Estrogen deficiency disrupts cerebrovascular regulation through multiple interconnected mechanisms, including downregulation of endothelial nitric oxide synthase (eNOS) signaling and upregulation of vasoconstrictive pathways (e.g., endothelin-1 [ET-1]), thereby impairing cerebrovascular vasomotor reactivity and promoting chronic hypoperfusion in regions critical for cognition, including the hippocampus, prefrontal cortex, and anterior cingulate cortex (Arjmand et al., 2024). These vascular changes may influence the homeostasis of neurodegeneration-related proteins, including amyloid-β and tau, through multiple potential pathways involving altered metabolism, production, release, or clearance; however, the specific mechanisms remain uncertain (Ekanayake et al., 2024; Dagum et al., 2026). Notably, estrogen-related mechanisms may contribute to cerebrovascular changes after menopause; however, evidence regarding menopausal hormone therapy (MHT) and cognitive outcomes remains mixed. Some large randomized trials, particularly among older postmenopausal women initiating hormone therapy later after menopause, reported increased dementia risk or no clear cognitive benefit, whereas recent large-scale meta-analytic evidence has suggested no consistent association between MHT use and the risk of MCI or dementia. This inconsistency highlights the complexity of estrogen-related mechanisms and cautions against assuming a direct causal relationship between estrogen replacement and reduced cognitive risk. Despite these mechanistic insights, there are currently no approved pharmacological treatments that reliably reverse cognitive impairment in MCI (Cepeda-Pineda et al., 2025). Disease-modifying anti-amyloid therapies are primarily indicated for early AD and are constrained by high cost, safety concerns, and potentially reduced efficacy in postmenopausal women, possibly due to underlying estrogen deficiency (Kurkinen, 2023; Qiao et al., 2023). These limitations highlight the need for safe, accessible, and feasible non-pharmacological strategies for this high-risk population.

Tai Chi, a traditional mind–body practice that integrates coordinated movement, controlled breathing, and focused attention, has been associated with improvements in cognitive function among older adults with MCI (Shao et al., 2024; Wen et al., 2025). Neuroimaging evidence further suggests that Tai Chi may enhance hippocampal gray matter volume and strengthen functional connectivity between the prefrontal cortex and hippocampus (Zhang et al., 2021b; Ben Ezzdine et al., 2025). However, prior randomized controlled trials have largely enrolled mixed-sex populations, without stratifying analyses by menopausal status or conducting dedicated trials in postmenopausal women, a distinct high-risk group. Moreover, substantial heterogeneity in intervention protocols and outcome measures persists, and the biological mechanisms underlying Tai Chi’s cognitive benefits remain insufficiently characterized. As a result, the potential contribution of estrogen deficiency-related cerebrovascular dysfunction to cognitive decline remains insufficiently understood. To date, few randomized controlled trials have examined whether Tai Chi may improve cognitive function in postmenopausal women with MCI in parallel with changes in cerebral perfusion and vascular function. In particular, the relationship between cerebrovascular regulation and circulating AD-related biomarkers remains underexplored in this population. Tai Chi combines low-intensity physical activity, controlled breathing, and mind–body practice and may therefore influence autonomic and vascular regulation. These effects may be relevant to cerebrovascular health and cerebral perfusion and, in turn, may contribute to cognitive function. This study was designed to address this gap by examining cognitive performance alongside cerebral perfusion, circulating vasomotor factors, and AD-related biomarkers.

To this end, we conducted a randomized controlled trial to evaluate the effects of a 12-week standardized 24-form Tai Chi program, compared with health education alone, on cognitive function and cerebrovascular health in postmenopausal women with MCI. We hypothesized that Tai Chi would lead to greater improvements in cognitive performance and cerebrovascular biomarkers than the control condition.

## 2 Materials and methods

### 2.1 Ethical approval and trial registration

The study was conducted in accordance with the Declaration of Helsinki (2013 revision) and reported in accordance with the CONSORT 2025 statement. Ethical approval was obtained from the Ethics Committee of the Second Affiliated Hospital of Fujian University of Traditional Chinese Medicine (Approval No. SPHFJP-K2022026-02; February 16, 2023). This prospective interventional study was registered with the Chinese Clinical Trial Registry (ChiCTR; registry URL: http://www.chictr.org.cn), with registration number ChiCTR2300068871, on March 2, 2023, prior to participant enrollment. Written informed consent was obtained from all participants. Participants were informed of their right to withdraw at any time without consequences for their routine medical care. The full trial protocol and prespecified statistical analysis plan (SAP) are available from the corresponding author upon reasonable request. No patients or members of the public were involved in the design, conduct, data analysis, interpretation of the findings, or preparation of the manuscript.

### 2.2 Study design and setting

This was a single-center, parallel-group, open-label randomized controlled trial with a 1:1 allocation ratio. The study was conducted at the Second Affiliated Hospital of Fujian University of Traditional Chinese Medicine (Fuzhou, Fujian Province, China) between June 2023 and September 2024. Eligible postmenopausal women with MCI were randomly assigned to either a Tai Chi intervention group or a health education control group for 12 weeks. Outcome assessments were performed by trained evaluators at two time points: baseline (within 7 days before the intervention) and post-intervention (within 14 days after completion of the 12-week program).

### 2.3 Study participants

#### 2.3.1 Recruitment

Participants were consecutively recruited from the Departments of Geriatrics and Neurology, as well as the Health Management Center of the study hospital. Initial screening, including cognitive assessment and medical history review, was conducted by trained research staff to determine eligibility.

#### 2.3.2 Eligibility criteria

Inclusion criteria were as follows: (1) postmenopausal women aged 50–75 years, defined as ≥ 12 consecutive months of amenorrhea without pathological cause, with no history of hysterectomy or bilateral oophorectomy, no hormone replacement therapy within the preceding 6 months, and no use of cognitive-enhancing herbal products or supplements within 3 months prior to enrollment; (2) diagnosis of MCI based on the Petersen criteria (2004) and the Chinese Guidelines for the Diagnosis and Treatment of Dementia and Cognitive Impairment (Writing Group of Chinese Guidelines for the Diagnosis and Treatment of Dementia and Cognitive Impairment, and Cognitive Disorders Professional Committee of the Neurologist Branch of Chinese Medical Doctor Association, 2018); although cerebrovascular findings identified on brain imaging were documented, they were not used to establish a separate diagnosis of vascular MCI, and no etiological classification as AD-related or vascular MCI was performed; (3) education-adjusted Montreal Cognitive Assessment (MoCA) score of 18–25 (Chinese version); (4) no contraindications to moderate-intensity exercise based on the screening assessment, and no regular structured exercise during the preceding 3 months, defined as fewer than 3 sessions per week and less than 30 min per session, as assessed using a study-specific structured self-report questionnaire developed by the research team and informed by relevant items from the International Physical Activity Questionnaire (IPAQ); and (5) provision of written informed consent.

Exclusion criteria included: (1) neurological disorders affecting cognitive function (e.g., viral encephalitis, Parkinson’s disease, or epilepsy); (2) severe psychiatric conditions that could interfere with study participation; (3) use of medications known to affect cognition within 3 months prior to enrollment; (4) severe organ dysfunction or confirmed malignancy; and (5) inability to complete the 12-week intervention or follow-up assessments.

As part of the eligibility screening and informed consent process, the study physician assessed each participant’s ability to understand the study purpose, procedures, potential risks and benefits, and to provide voluntary written informed consent.

#### 2.3.3 Sample size calculation

The sample size was estimated based on the primary outcome, defined as the change in MoCA total score from baseline to post-intervention. The calculation assumed a two-sided significance level (α) of 0.05, 80% power (1-β), a between-group difference of 2.5 points, and a standard deviation of 2.8 points. The effect-size assumptions were partly informed by data from an unpublished pilot study conducted by our research team and were considered consistent with prior high-quality Tai Chi trials in populations with MCI (Li et al., 2025a; Li et al., 2025b). This yielded a required minimum sample size of 17 participants per group. Allowing for a 15% attrition rate, we aimed to recruit 20 participants per group (total *n* = 40). Sample size calculations were performed using PASS version 15.0.

### 2.4 Randomization, allocation concealment, and blinding

Participants were randomly assigned in a 1:1 ratio to either the Tai Chi group or the health education control group. Block randomization with a block size of four was used to ensure balance between groups. The allocation sequence was generated using SPSS version 25.0 by an independent biostatistician who was not involved in participant recruitment, intervention delivery, or outcome assessment.

Allocation concealment was maintained through sequentially numbered, opaque, sealed envelopes, which were opened only after completion of baseline assessments and informed consent procedures. The personnel responsible for participant enrollment and intervention assignment had no access to the allocation sequence before the corresponding envelope was opened. Due to the nature of the intervention, blinding of participants and instructors was not feasible. However, outcome assessors, magnetic resonance imaging (MRI) readers, laboratory personnel, and the statistician remained blinded to group allocation throughout the study. Participants were instructed not to disclose their group assignment during assessments.

### 2.5 Intervention protocols

#### 2.5.1 Common intervention for both groups

All participants received standardized education on MCI prevention and management over 12 weeks. This consisted of two sessions per week (≥ 15 min each), delivered as in-person lectures or online modules, covering evidence-based strategies for cognitive training, as well as diet and lifestyle modifications to support cognitive health. Monthly telephone follow-up calls were conducted throughout the intervention period to monitor adherence. Participants were asked to maintain their usual daily activities and to refrain from initiating any additional structured exercise programs or cognitive interventions during the study period.

The health education control group was selected as a pragmatic, non-exercise comparator to provide participants with general health-related information while avoiding a structured exercise intervention. This comparator was intended to provide a non-exercise reference condition rather than an active control matched for contact time, supervision, or physical activity.

#### 2.5.2 Tai Chi intervention

In addition to the standardized education, participants in the intervention group underwent a 12-week supervised program of 24-form simplified Tai Chi (Developed by the General Administration of Sport of China, 1956). Training was conducted three times per week, with sessions spaced 1–2 days apart, and each session lasting 60 min. Each session comprised a 5-min standardized warm-up (including neck mobility, shoulder rotations, trunk movements, and lower-limb joint mobilization), a 50-min practice of the 24-form Tai Chi sequence under instructor supervision, and a 5-min cool-down period involving static stretching of major muscle groups (quadriceps, hamstrings, gastrocnemius, soleus, and gluteal muscles).

All training sessions were conducted in a dedicated indoor facility and led by physicians with at least 2 years of experience teaching the 24-form Tai Chi, following a standardized intervention manual. Exercise intensity was maintained at a moderate level (40–60% of age-predicted maximum heart rate, corresponding to a Borg Rating of Perceived Exertion of 11–13) and monitored continuously using wrist-worn heart rate devices. Intervention fidelity was supported through strict adherence to the protocol and real-time, individualized correction of movement technique. Attendance was recorded for every session. Participants who missed a session were offered a one-on-one make-up session within 48 h. Adherence was defined *a priori* as attendance at ≥ 80% of sessions.

#### 2.5.3 Safety monitoring

All adverse events (AEs), including falls, muscle strain, joint pain, and cardiovascular symptoms, were systematically screened, documented, and graded by severity as mild, moderate, or severe according to the Common Terminology Criteria for Adverse Events (CTCAE) version 5.0. For each event, the time of onset, severity grade, duration, suspected relationship to the intervention, and management were recorded. AEs were managed promptly by the study medical team. Serious adverse events were reported to the ethics committee within 24 h.

### 2.6 Outcome measures

#### 2.6.1 Primary outcome

The primary outcome was the change in global cognitive function, assessed by the total score on the validated Chinese version of the MoCA. The MoCA is a widely used screening tool for MCI that evaluates seven cognitive domains: visuospatial/executive function, naming, attention, language, abstraction, delayed recall, and orientation (maximum score = 30). Nevertheless, it is a brief cognitive screening instrument designed for rapid assessment of cognitive impairment in clinical and research settings. It does not provide the breadth and depth of assessment offered by a comprehensive neuropsychological test battery, nor does it cover all cognitive domains with comparable depth. In this study, the MoCA total score was used as a pragmatic measure of overall cognitive performance rather than as a comprehensive measure of global cognitive function. The domain-specific scores were interpreted as screening-level indicators of performance in the corresponding cognitive domains rather than as comprehensive domain-specific neuropsychological measures. Scores were adjusted for education by adding one point for participants with fewer than 11 years of formal education. The same MoCA version was administered at baseline and after the 12-week intervention; no alternative or parallel version was used. All assessors underwent standardized training and achieved high inter-rater reliability, with an intraclass correlation coefficient (ICC) of 0.92 for total scores. Although the MoCA was originally developed primarily as a cognitive screening instrument, it has also been used as a measure of global cognitive function to assess longitudinal changes in intervention studies involving individuals with MCI (Krishnan et al., 2017; Han et al., 2023).

#### 2.6.2 Secondary outcomes

Secondary outcomes included domain-specific cognitive performance, CBF, circulating vasomotor factors, and AD-related biomarkers. Domain-specific cognitive function was assessed by analyzing scores across the seven individual MoCA domains. Regional and global CBF were measured using a 3.0 T MRI scanner (Signa HDX, GE Healthcare, Milwaukee, WI, USA) with a pseudo-continuous arterial spin labeling (pCASL) sequence. Detailed acquisition parameters, region of interest (ROI) delineation procedures, and quality-control protocols are provided in Supplementary Method S1. Predefined ROIs in the bilateral middle frontal gyrus, anterior cingulate cortex, hippocampus, and parahippocampal gyrus were independently delineated by two board-certified radiologists blinded to group allocation. Each ROI was measured in triplicate, and the mean value was used for analysis. Inter-rater and intra-rater reliability were assessed using intraclass correlation coefficients (ICCs) and coefficients of variation (CVs). These analyses assessed the within-dataset repeatability and inter-rater reproducibility of ROI-based CBF measurements derived from the same ASL acquisition, rather than test–retest stability of CBF measurements across repeated MRI acquisitions at different time points. Intra-rater ICCs ranged from 0.89 to 0.94, inter-rater ICCs from 0.85 to 0.91, and CVs from 3.2 to 5.7%.

Circulating vasomotor factors were assessed using fasting venous blood samples collected after an overnight fast of at least 8 h. Samples were centrifuged at 3000 rpm for 10 min at 4 °C within one hour of collection, and plasma and serum aliquots were stored at −80 °C until analysis. Plasma nitric oxide (NO) levels were determined using the Griess colorimetric method, while plasma ET-1, prostacyclin (PGI_2_), and thromboxane A_2_ (TXA_2_) were measured using commercially available enzyme-linked immunosorbent assay (ELISA) kits according to the manufacturers’ protocols. All assays demonstrated intra-assay coefficients of variation below 5% and inter-assay coefficients below 8%. The ET-1/NO ratio and TXA_2_/PGI_2_ were calculated as indices of systemic vasomotor balance.

Serum concentrations of AD-related biomarkers, including total tau (T-tau), phosphorylated tau (P-tau), and amyloid-β_1-42_ (Aβ_1-42_), were also quantified using ELISA.

#### 2.6.3 Baseline and safety outcomes

Baseline characteristics included age, body mass index (BMI), years of education, duration of menopause, and comorbidities (hypertension, diabetes, and silent brain infarction). The safety outcome was the incidence of adverse events (AEs) during the 12-week intervention period.

### 2.7 Statistical analysis

All statistical analyses were performed using SPSS version 25.0 (IBM Corp., Armonk, NY, USA). A two-sided *p* < 0.05 was considered statistically significant, except where adjusted thresholds were applied for multiple comparisons. Normality of continuous variables was assessed using the Shapiro–Wilk test and visual inspection of distribution plots. Baseline characteristics were summarized as mean ± standard deviation (SD) for normally-distributed variables, median (P_25_, P_75_) for non-normally-distributed continuous variables, and counts (percentages) for categorical variables. All analyses were performed in the intention-to-treat (ITT) population; there were no missing data because all randomized participants completed the 12-week follow-up.

Linear mixed-effects models (LMMs) were used as the primary analytical approach for all longitudinal outcomes. Separate models were fitted for each outcome, with group, time, and the group-by-time interaction as fixed effects and participant-specific random intercepts to account for within-participant correlations. The group-by-time interaction was used as the primary test of the intervention effect, representing the between-group difference in changes from baseline to post-intervention. Estimated *β* coefficients with 95% confidence intervals (CIs) and corresponding *p* values were reported. Model assumptions were evaluated using residual diagnostics. Cohen’s *d* was calculated from between-group differences in change scores, with 95% CIs estimated using the noncentral *t* distribution. Effect sizes of 0.2, 0.5, and 0.8 were interpreted as small, medium, and large effects, respectively.

Baseline-adjusted analysis of covariance (ANCOVA) was performed as a sensitivity analysis for outcomes meeting the relevant model assumptions. Post-intervention outcomes were specified as dependent variables, treatment group as the fixed factor, and the corresponding baseline outcomes as the covariate. The homogeneity-of-regression-slopes assumption was evaluated before ANCOVA. For outcomes where the homogeneity-of-regression-slopes assumption was violated, conventional baseline-adjusted ANCOVA was not performed. Instead, baseline-adjusted linear models including the group × baseline interaction were fitted as sensitivity analyses. Simple-slopes analyses were performed at the 25th percentile, median, and 75th percentile of baseline values to estimate between-group differences in post-intervention outcomes across representative baseline levels. The LMM remained the primary longitudinal analysis because the group-by-time interaction directly estimates the between-group difference in change from baseline to post-intervention.

Supplementary analyses examined within-group and between-group changes using change scores (post-intervention minus baseline). Paired *t*-tests or Wilcoxon signed-rank tests were used for within-group comparisons, and independent-samples *t*-tests or Mann–Whitney *U* tests for between-group comparisons, as appropriate.

For the seven exploratory MoCA domain-specific analyses, a Bonferroni correction was applied, yielding an adjusted significance threshold of *p* < 0.007 (0.05/7). Exploratory Spearman rank correlation analyses assessed associations between changes in MoCA total score and six prespecified biomarkers (global mean CBF, plasma NO and ET-1, and serum T-tau, P-tau, and Aβ_1-42_). Correlation coefficients with 95% CIs were reported. A separate Bonferroni-adjusted significance threshold of *p* < 0.008 (0.05/6) was applied. These analyses were considered exploratory and hypothesis-generating.

## 3 Results

### 3.1 Participant flow

Between June 2023 and September 2024, a total of 56 postmenopausal women with suspected MCI were screened for eligibility. 16 participants were excluded: 5 did not meet inclusion criteria (MoCA total score outside the predefined range, *n* = 3; ineligible menopausal status, *n* = 2), 6 met exclusion criteria (hormone replacement therapy, *n* = 2; regular long-term exercise, *n* = 3; unstable chronic medical conditions, *n* = 1), and 5 declined to participate. The remaining 40 participants were randomized 1:1 to the Tai Chi group (*n* = 20) or the health education control group (*n* = 20). All randomized participants completed the 12-week intervention and post-intervention assessments, with no withdrawals or loss to follow-up. Accordingly, the intention-to-treat and per-protocol populations were identical (Figure 1).

**Figure 1:**
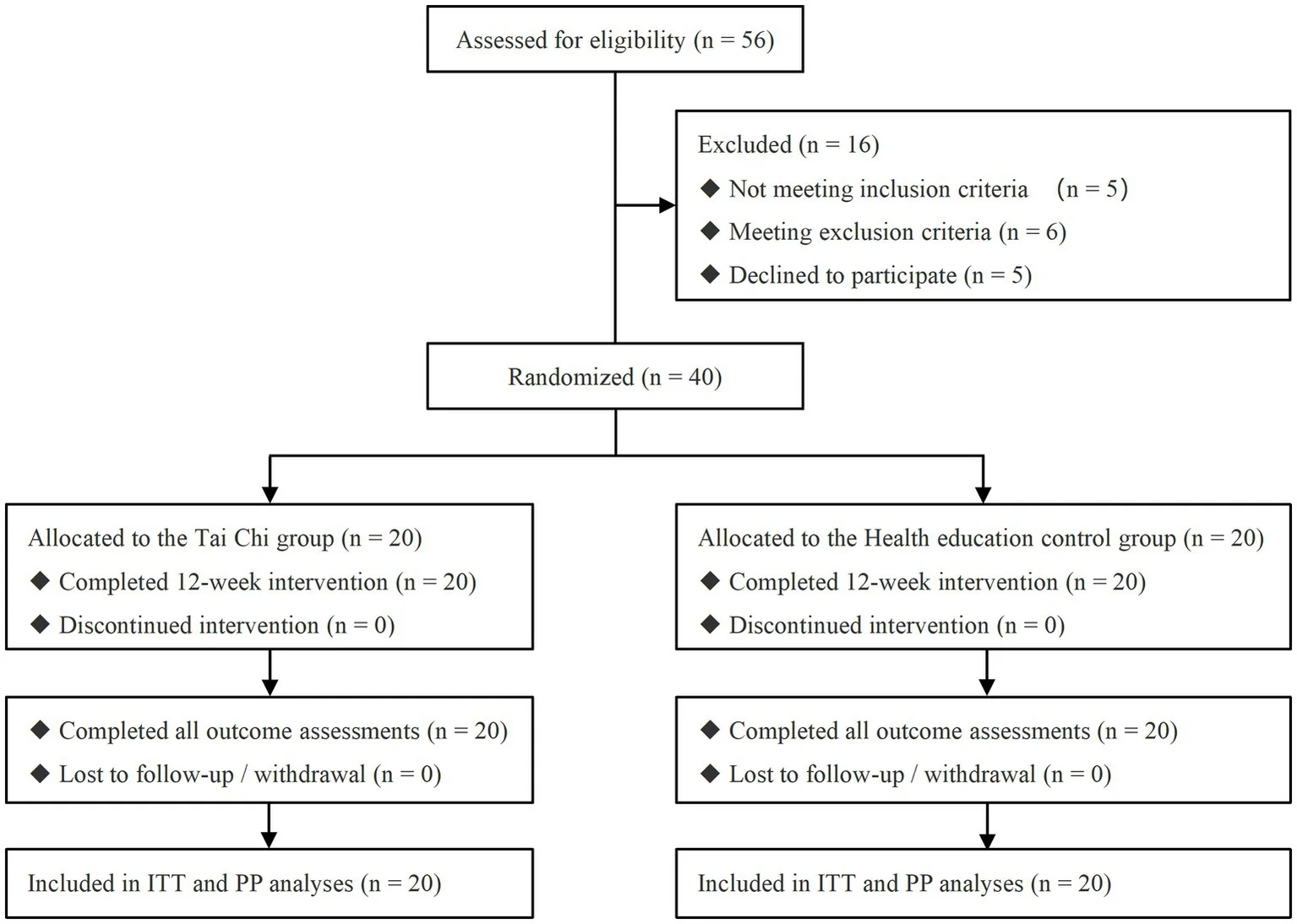
Flow chart of participant recruitment, randomization, and follow-up. ITT, intention-to-treat; PP, per-protocol.

### 3.2 Baseline characteristics

Baseline demographic and clinical characteristics, including age, BMI, years of education, menopause duration, and comorbidity profiles, are presented descriptively for the two groups (Table 1). No formal between-group significance testing was performed in accordance with recommendations for randomized controlled trials. Baseline values for MoCA scores, global and regional CBF, circulating vasomotor factors, and serum AD-related biomarkers are likewise presented descriptively in Tables 2–4.

**Table 1:** Baseline characteristics of participants in the two groups.

Characteristic	Health education control group (*n* = 20)	Tai Chi group (*n* = 20)
Age (years)	59.20 ± 5.24	59.00 ± 5.52
BMI (kg/m^2^)	22.61 ± 2.26	21.92 ± 2.61
Years of Education	12.00 (9.00, 14.50)	12.50 (9.75, 15.00)
Menopause duration (years)	9.60 ± 4.49	9.25 ± 5.42
Comorbidity		
Hypertension (%)	6 (30.00)	7 (35.00)
Diabetes Mellitus (%)	0 (0.00)	3 (15.00)
Silent Brain Infarction (%)	18 (90.00)	18 (90.00)

Normally distributed continuous variables are presented as mean ± SD; non-normally distributed variables as median (P_25_, P_75_). Baseline characteristics were summarized descriptively, and no formal statistical comparisons between groups were performed because randomization was used to achieve baseline balance by design. BMI, body mass index; SD, standard deviation.

**Table 2:** Linear mixed-effects model analysis of changes in MoCA scores during the 12-week intervention.

Cognitive domain	Health education control group (*n* = 20)		Tai Chi group (*n* = 20)		Group-by-time interaction *β* (95% CI)	*p*-value	Cohen’s *d* [95% CI]
	Baseline	Post-intervention	Baseline	Post-intervention			
MoCA Total Score	25.00 (23.00, 25.00)	24.50 (20.00, 25.75)	25.00 (24.00, 25.00)	28.00 (27.00, 29.00)	4.95 (3.98, 5.92)	**< 0.001**	3.28 [2.32, 4.24]
Visuospatial/Executive function	3.50 (3.00, 4.00)	3.00 (3.00, 4.00)	4.00 (3.00, 4.00)	5.00 (5.00, 5.00)	1.10 (0.69, 1.51)	**< 0.001**	1.71 [0.98, 2.44]
Naming	3.00 (3.00, 3.00)	3.00 (2.00, 3.00)	3.00 (3.00, 3.00)	3.00 (3.00, 3.00)	0.45 (0.12, 0.78)	0.010	0.71 [0.07, 1.35]
Attention	5.00 (4.25, 5.00)	5.00 (4.00, 6.00)	5.00 (5.00, 5.00)	6.00 (6.00, 6.00)	1.05 (0.60, 1.50)	**< 0.001**	1.50 [0.80, 2.21]
Language	2.00 (1.25, 3.00)	2.50 (1.00, 3.00)	2.00 (1.25, 3.00)	3.00 (2.00, 3.00)	0.40 (0.04, 0.76)	0.030	0.71 [0.07, 1.35]
Abstraction	1.00 (1.00, 2.00)	1.00 (1.00, 2.00)	2.00 (1.00, 2.00)	2.00 (2.00, 2.00)	0.25 (−0.11, 0.61)	0.170	0.44 [−0.19, 1.07]
Delayed Recall	3.00 (3.00, 4.00)	3.00 (3.00, 4.00)	3.00 (3.00, 3.00)	5.00 (4.00, 5.00)	1.50 (1.13, 1.87)	**< 0.001**	2.60 [1.75, 3.45]
Orientation	6.00 (6.00, 6.00)	6.00 (5.25, 6.00)	6.00 (6.00, 6.00)	6.00 (6.00, 6.00)	0.20 (−0.09, 0.49)	0.165	0.45 [−0.18, 1.08]

Data are presented as median (P_25_, P_75_), whereas intervention effects were estimated using linear mixed-effects models. Models included group, time, and group-by-time interaction as fixed effects, with participant-specific random intercepts. The group-by-time interaction represents the between-group difference in change from baseline to post-intervention and was considered the primary test of the intervention effect. β represents the estimated difference in change between the Tai Chi and health education control groups, with positive values indicating greater improvement in the Tai Chi group. Cohen’s d was calculated from individual pre-post change scores, and 95% confidence intervals were estimated using the noncentral t distribution. Magnitude thresholds for Cohen’s d: 0.2 = small, 0.5 = medium, 0.8 = large. Bold *p*-values indicate statistically significant results.” here, immediately prior to the existing description of significance thresholds for the primary outcome and domain-specific analyses. The MoCA total score was considered the primary outcome (α = 0.05). For the seven MoCA domain-specific analyses, statistical significance was defined as *p* < 0.007 after Bonferroni correction.

MoCA, Montreal Cognitive Assessment; CI, confidence interval.

**Table 3:** Linear mixed-effects model analysis of changes in CBF of cognition-related brain regions during the 12-week intervention.

Brain Region	Health education control group (*n* = 20)		Tai Chi group (*n* = 20)		Group-by-Time Interaction *β* (95% CI)	*P*-value	Cohen’s *d* [95% CI]
	Baseline	Post-intervention	Baseline	Post-intervention			
Left Middle Frontal Gyrus	52.32 ± 4.99	47.86 ± 5.64	51.01 ± 4.16	57.95 ± 4.93	11.39 (8.09, 14.69)	**< 0.001**	2.21 [1.41, 3.00]
Right Middle Frontal Gyrus	51.78 ± 4.89	47.74 ± 5.46	50.88 ± 3.58	58.75 ± 6.22	11.91 (8.60, 15.21)	**< 0.001**	2.31 [1.51, 3.10]
Left Anterior Cingulate Cortex	51.81 ± 3.46	48.44 ± 3.57	50.92 ± 4.75	60.99 ± 6.45	13.44 (9.72, 17.16)	**< 0.001**	2.32 [1.51, 3.13]
Right Anterior Cingulate Cortex	51.60 ± 3.60	48.64 ± 3.40	50.26 ± 4.93	60.31 ± 6.28	13.02 (9.82, 16.21)	**< 0.001**	2.61 [1.76, 3.46]
Left Hippocampus	49.34 ± 3.79	46.82 ± 3.81	48.80 ± 3.74	52.85 ± 4.07	6.58 (3.95, 9.21)	**< 0.001**	1.60 [0.89, 2.32]
Right Hippocampus	49.02 ± 4.47	46.47 ± 4.24	49.00 ± 3.71	54.47 ± 4.36	8.02 (5.11, 10.94)	**< 0.001**	1.77 [1.03, 2.50]
Left Parahippocampal Gyrus	50.61 ± 3.18	46.46 ± 3.40	49.50 ± 3.04	60.12 ± 5.60	14.78 (12.06, 17.50)	**< 0.001**	3.48 [2.48, 4.48]
Right Parahippocampal Gyrus	51.48 ± 4.33	47.45 ± 2.98	49.58 ± 3.53	60.91 ± 6.79	15.36 (12.00, 18.72)	**< 0.001**	2.93 [2.02, 3.83]
Global mean CBF	51.58 ± 3.74	46.58 ± 4.23	49.84 ± 3.87	60.13 ± 4.36	15.29 (12.58, 17.99)	**< 0.001**	3.62 [2.60, 4.64]

All CBF values are presented as mean ± SD, with the unit of mL·100 g^−1^·min^−1^. The linear mixed-effects models included group, time, and group-by-time interaction as fixed effects, with participant-specific random intercepts. The group-by-time interaction represents the between-group difference in change from baseline to post-intervention and was considered the primary test of the intervention effect. β represents the estimated difference in change between the Tai Chi and health education control groups, with positive values indicating greater improvement in the Tai Chi group. Cohen’s d was calculated from individual pre-post change scores, and 95% confidence intervals were estimated using the noncentral t distribution. Magnitude thresholds for Cohen’s d: 0.2 = small, 0.5 = medium, 0.8 = large.

CBF, cerebral blood flow; SD, standard deviation.

Bold *p*-values indicate statistically significant results (*p* < 0.05).

**Table 4:** Linear mixed-effects model analysis of changes in circulating vasomotor factors and serum AD-related biomarkers during the 12-week intervention.

Outcome	Health education control group (*n* = 20)		Tai Chi group (*n* = 20)		Group-by-time interaction *β* (95% CI)	*P*-value	Cohen’s *d* [95% CI]
	Baseline	Post-intervention	Baseline	Post-intervention			
NO (μmol/L)	49.74 ± 6.37	49.72 ± 7.84	49.13 ± 3.71	59.52 ± 5.51	10.41 (5.02, 15.79)	**< 0.001**	1.21 [0.54, 1.89]
ET-1 (pg/mL)	159.91 ± 28.93	110.34 ± 33.28	171.73 ± 34.00	93.13 ± 34.59	−29.04 (−56.59, −1.48)	**0.039**	−0.67 [−1.31, −0.03]
PGI_2_ (pg/mL)	79.95 ± 14.46	189.77 ± 28.13	85.87 ± 17.00	178.29 ± 36.96	−17.39 (−37.67, 2.89)	0.091	−0.55 [−1.18, 0.08]
TXA_2_ (pg/mL)	251.05 ± 42.53	183.01 ± 43.06	236.22 ± 37.21	169.93 ± 44.98	1.74 (−35.90, 39.39)	0.926	0.03 [−0.59, 0.65]
ET-1/NO	3.28 ± 0.82	2.32 ± 0.92	3.50 ± 0.67	1.56 ± 0.54	−0.97 (−1.61, −0.34)	**0.004**	−0.98 [−1.64, −0.32]
TXA_2_/PGI_2_	3.25 ± 0.81	1.00 ± 0.34	2.85 ± 0.73	1.01 ± 0.37	0.40 (−0.06, 0.85)	0.084	0.56 [−0.07, 1.19]
T-tau (pg/mL)	644.52 ± 125.65	670.75 ± 118.32	685.19 ± 123.95	455.97 ± 90.88	−255.45 (−349.12, −161.78)	**< 0.001**	−1.75 [−2.49, −1.01]
P-tau (pg/mL)	581.06 ± 94.67	536.72 ± 101.11	599.91 ± 89.09	419.89 ± 91.90	−135.67 (−201.68, −69.66)	**< 0.001**	−1.32 [−2.01, −0.63]
Aβ_1-42_ (pg/mL)	707.61 ± 96.85	683.72 ± 110.80	678.94 ± 127.94	485.63 ± 104.38	−169.41 (−255.73, −83.08)	**< 0.001**	−1.26 [−1.94, −0.58]

Data are presented as mean ± SD. The linear mixed-effects models included group, time, and group-by-time interaction as fixed effects, with participant-specific random intercepts. The group-by-time interaction represents the between-group difference in change from baseline to post-intervention and was considered the primary test of the intervention effect. β represents the estimated between-group difference in change (Tai Chi minus health education control group), with positive values indicating a larger increase (or a smaller decrease) in the Tai Chi group, whereas negative values indicating a larger decrease (or a smaller increase) in the Tai Chi group. Cohen’s d was calculated from individual pre-post change values, and 95% confidence intervals were estimated using the noncentral t distribution. Magnitude thresholds for Cohen’s d: 0.2 = small, 0.5 = medium, 0.8 = large.

Bold *p*-values indicate statistically significant results (*p* < 0.05).

NO, nitric oxide; ET-1, endothelin-1; PGI_2_, prostacyclin; TXA_2_, thromboxane A_2_; T-tau, total tau; P-tau, phosphorylated tau; Aβ_1-42_, amyloid-beta_1–42_; SD, standard deviation.

### 3.3 Primary outcome: overall cognitive performance assessed by the MoCA

The primary LMM demonstrated a significant group-by-time interaction for the MoCA total score (*β* = 4.95, 95% CI [3.98, 5.92]; *p* < 0.001), indicating greater improvement in overall cognitive performance as assessed by the MoCA from baseline to post-intervention in the Tai Chi group than in the health education control group. The corresponding Cohen’s *d* was 3.28 (95% CI [2.32, 4.24]) (Table 2; Figure 2).

**Figure 2:**
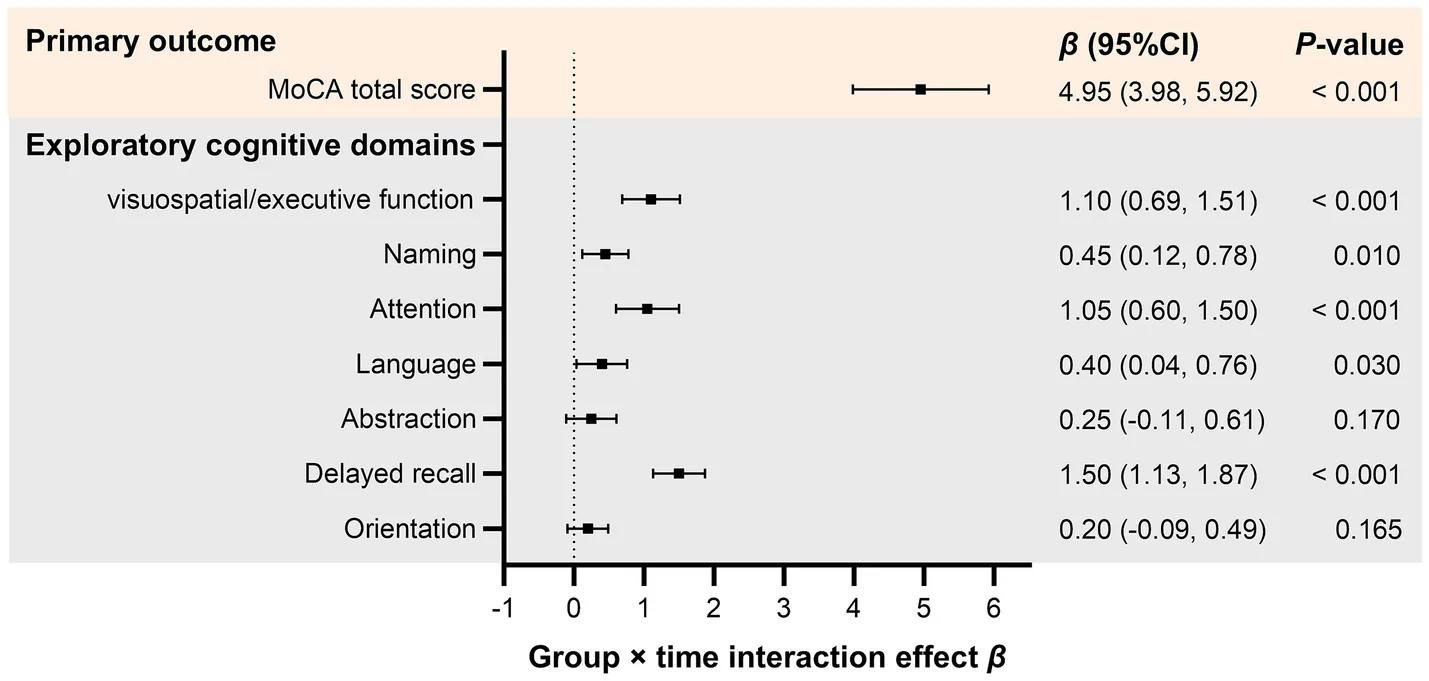
Forest plot of group-by-time interaction effects on MoCA total and domain-specific scores. Note: Each square represents the estimated regression coefficient (*β*) for the group-by-time interaction from the linear mixed-effects model (LMM), and horizontal bars indicate the corresponding 95% confidence intervals (CIs). The vertical dashed line at *β* = 0 represents the null effect. The primary outcome (MoCA total score) is shown separately from the seven exploratory cognitive domains. The MoCA total score was assessed using a two-sided significance threshold of *p* < 0.05, whereas *p* values for the seven domain-specific analyses were adjusted for multiple comparisons using the Bonferroni method, yielding a corrected significance threshold of *p* < 0.007. *p* values shown in the plot are unadjusted values; statistical significance for the domain-specific analyses was determined using the Bonferroni-adjusted threshold. Numerical estimates of *β*, 95% CIs, and corresponding *p* values are shown on the right. MoCA, Montreal Cognitive Assessment.

Among the seven exploratory MoCA domain scores, group-by-time interactions remained statistically significant after Bonferroni correction (*p* < 0.007) for visuospatial/executive function (*β* = 1.10, 95% CI [0.69, 1.51]), attention (*β* = 1.05, 95% CI [0.60, 1.50]), and delayed recall (*β* = 1.50, 95% CI [1.13, 1.87]) (all *p* < 0.001). Corresponding Cohen’s *d* values were 1.71 (95% CI [0.98, 2.44]), 1.50 (95% CI [0.80, 2.21]), and 2.60 (95% CI [1.75, 3.45]), respectively. No significant group-by-time interactions were detected for naming, language, abstraction and orientation domains at the multiplicity-adjusted threshold (Table 2; Figure 2).

Estimated marginal mean (EMM) trajectories showed greater increases from baseline to post-intervention in the MoCA total score and in delayed recall, visuospatial/executive function, and attention scores in the Tai Chi group than in the health education control group (Figure 3).

**Figure 3:**
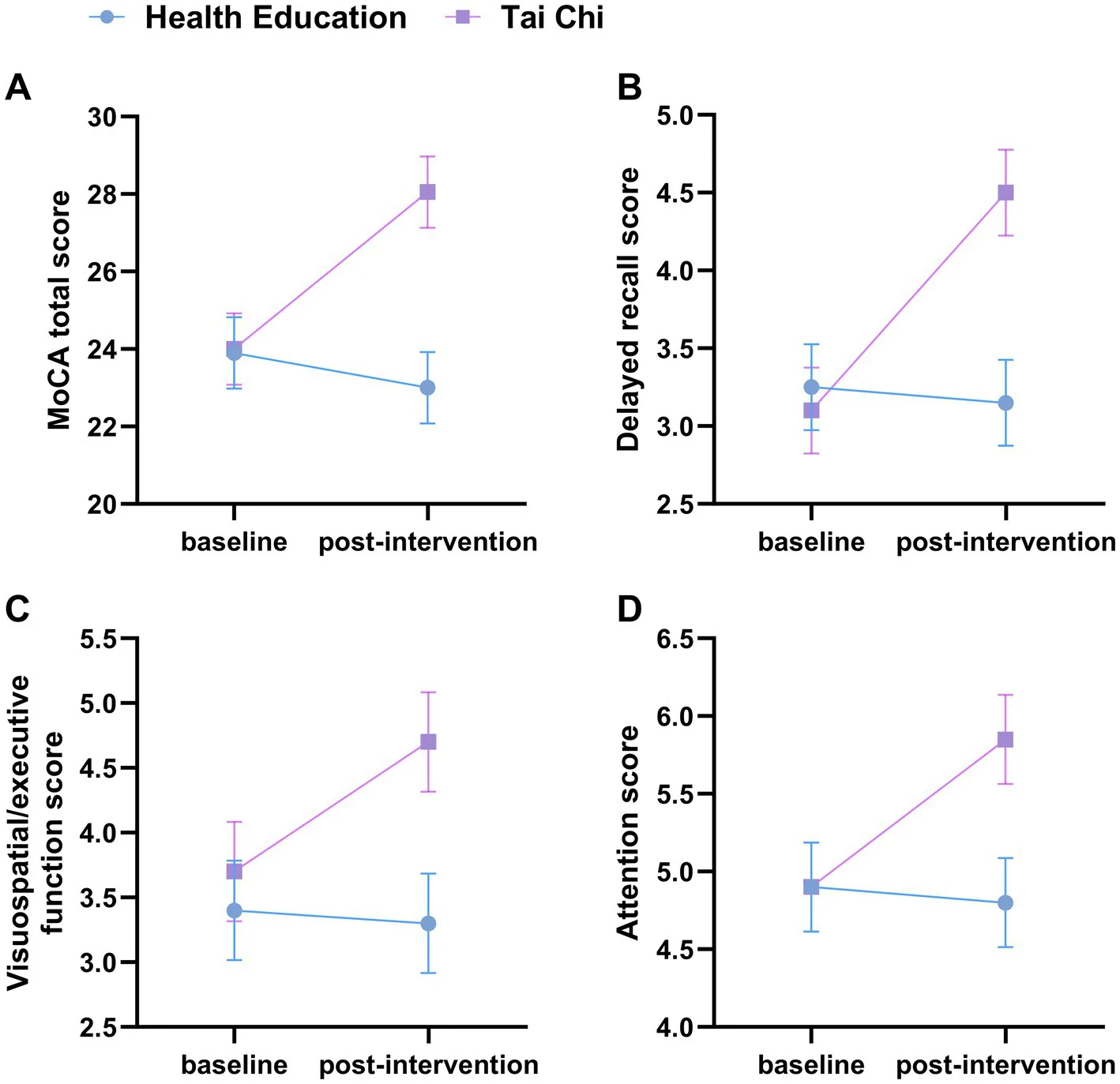
Estimated marginal mean trajectories of MoCA total and domain-specific scores in the Tai Chi and health education control groups. Note: Estimated marginal means (EMMs) and 95% confidence intervals (CIs) were derived from linear mixed-effects models with group, time, and group-by-time interaction as fixed effects and participant-specific random intercepts. Subplots show changes from baseline to post-intervention in **(A)** MoCA total score, **(B)** delayed recall score, **(C)** visuospatial/executive function score, and **(D)** attention score. Blue circles indicate the health education control group, and purple squares indicate the Tai Chi group. All four outcomes showed significant group-by-time interactions (all *p* < 0.001), with greater improvements in the Tai Chi group than in the health education control group. MoCA, Montreal Cognitive Assessment.

For the MoCA total score, an additional baseline-by-group interaction model yielded a significant interaction between baseline MoCA score and treatment group (*β* = −0.69, 95% CI [−1.28, −0.10]; *p* = 0.023), indicating that the magnitude of the between-group difference at post-intervention varied according to baseline MoCA score (Supplementary Table S2). Simple-slopes analyses showed significant between-group differences in post-intervention MoCA scores at the 25th percentile, median, and 75th percentile of baseline MoCA values, with adjusted mean differences of 5.58 (95% CI [4.53, 6.62]), 4.92 (95% CI [4.03, 5.81]), and 4.19 (95% CI [3.11, 5.28]), respectively (all *p* < 0.001; Supplementary Table S3). Thus, although the magnitude of the estimated between-group difference varied with baseline cognitive status, its direction and statistical significance were maintained across the selected baseline levels.

Supplementary between-group analyses of change scores yielded results broadly consistent with the primary LMM findings (Supplementary Table S4), providing complementary evidence for the observed between-group differences. Individual-level distributions of changes from baseline to post-intervention in the MoCA total score and the three MoCA domain scores showing significant group-by-time interactions are presented in Supplementary Figure S1.

### 3.4 Secondary outcomes

#### 3.4.1 Cerebral blood flow

LMMs revealed significant group-by-time interactions for global mean CBF and CBF in all eight predefined cognition-related brain regions (all *p* < 0.001) (Table 3; Figure 4). Global mean CBF increased significantly more in the Tai Chi group than in the health education control group (*β* = 15.29, 95% CI [12.58, 17.99]; *p* < 0.001; Cohen’s *d* = 3.62, 95% CI [2.60, 4.64]). Descriptively, global mean CBF increased from 49.84 ± 3.87 to 60.13 ± 4.36 mL·100 g^−1^·min^−1^ in the Tai Chi group, whereas it decreased from 51.58 ± 3.74 to 46.58 ± 4.23 mL·100 g^−1^·min^−1^ in the health education control group.

**Figure 4:**
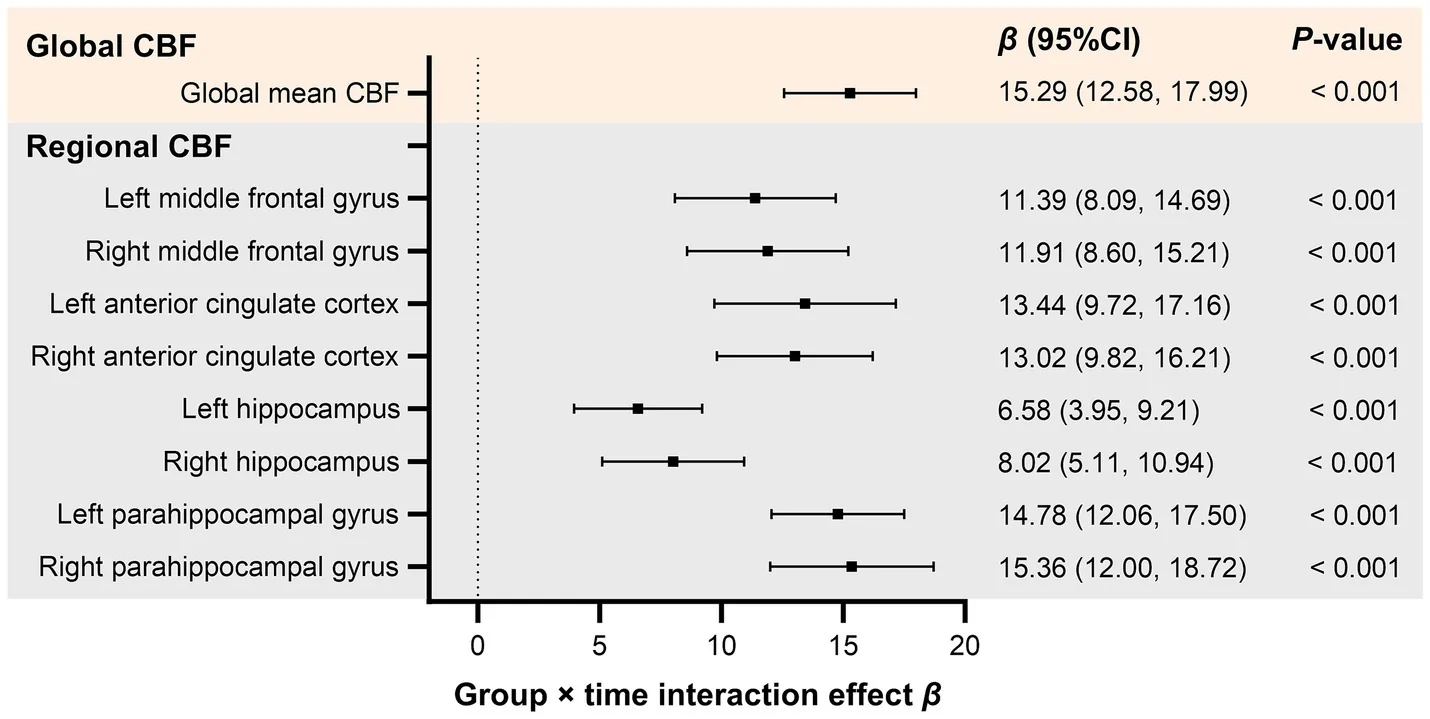
Forest plot of group-by-time interaction effects on global and regional CBF. Note: Each square represents the estimated regression coefficient (*β*) for the group-by-time interaction from the linear mixed-effects model (LMM), and horizontal bars indicate the corresponding 95% confidence intervals (CIs). The vertical dashed line at *β* = 0 represents the null effect. All group-by-time interaction effects were statistically significant (all *p* < 0.001). Numerical estimates of *β*, 95% CIs, and corresponding *p* values are shown on the right. CBF, cerebral blood flow.

Significant group-by-time interaction effects were also observed across all predefined regional CBF measures, with *β* estimates ranging from 6.58 to 15.36 and Cohen’s *d* values ranging from 1.60 to 3.48. The largest standardized effects were observed in the left and right parahippocampal gyrus (Cohen’s *d* = 3.48 and 2.93, respectively) (Table 3; Figure 4).

EMM trajectories showed greater increases in global and regional CBF from baseline to post-intervention in the Tai Chi group than in the health education control group (Figure 5).

**Figure 5:**
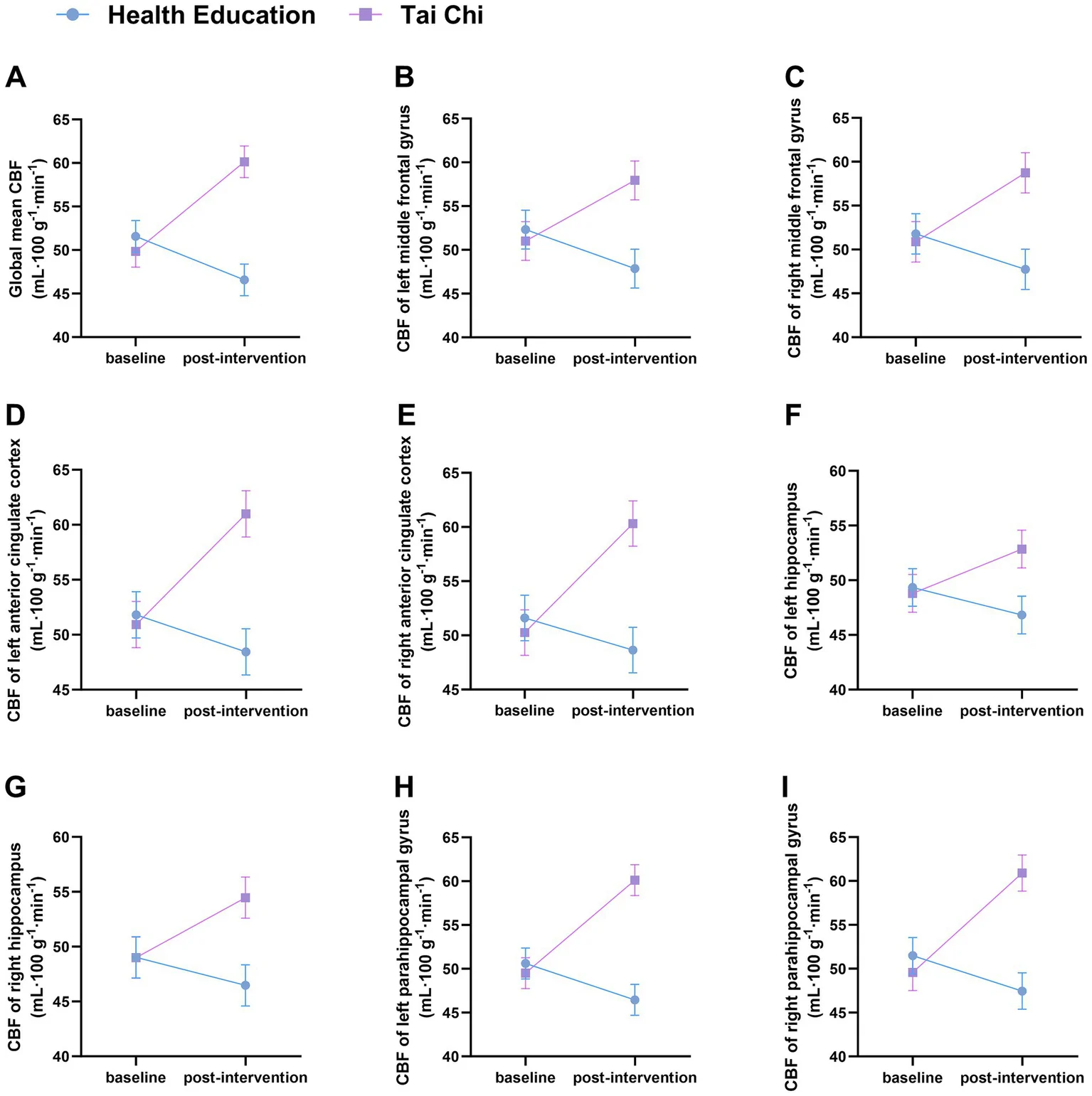
Estimated marginal mean trajectories of global and regional CBF in the Tai Chi and health education control groups. Note: Estimated marginal means (EMMs) and 95% confidence intervals (CIs) were derived from linear mixed-effects models with group, time, and group-by-time interaction as fixed effects and participant-specific random intercepts. Subplots show changes from baseline to post-intervention in **(A)** Global mean CBF, **(B)** left middle frontal gyrus, **(C)** right middle frontal gyrus, **(D)** left anterior cingulate cortex, **(E)** right anterior cingulate cortex, **(F)** left hippocampus; **(G)** right hippocampus; **(H)** left parahippocampal gyrus, and **(I)** right parahippocampal gyrus. Blue circles indicate the health education control group, and purple squares indicate the Tai Chi group. All nine outcomes showed significant group-by-time interactions (all *p* < 0.001), with greater increases in CBF from baseline to post-intervention in the Tai Chi group than in the health education control group. CBF, cerebral blood flow.

For global mean CBF, the baseline-by-group interaction model also showed a significant interaction between baseline CBF and treatment group (β = 0.70, 95% CI [0.06, 1.33]; *p* = 0.032; Supplementary Table S2). Simple-slopes analyses demonstrated significant between-group differences in post-intervention global mean CBF at the 25th percentile, median, and 75th percentile of baseline CBF, with adjusted mean differences of 12.84 (95% CI [10.01, 15.67]), 14.43 (95% CI [12.02, 16.85]), and 16.68 (95% CI [13.53, 19.83]), respectively (all *p* < 0.001; Supplementary Table S3). Thus, although the magnitude of the between-group difference increased with higher baseline CBF, the direction and statistical significance of the effect were maintained across the selected baseline levels.

Supplementary between-group analyses of change scores showed results broadly consistent with the primary LMM findings (Supplementary Table S5). Individual-level distributions of changes from baseline to post-intervention in global mean CBF and CBF in cognition-related brain regions are presented in Supplementary Figure S2.

#### 3.4.2 Circulating vasomotor factors

LMMs identified significant group-by-time interactions for plasma NO, ET-1, and the ET-1/NO ratio (*p* < 0.001, 0.039, and 0.004, respectively), but not for PGI2, TXA2, and the TXA2/PGI2 ratio (Table 4; Figure 6). NO showed a significantly greater increase in the Tai Chi group than in the health education control group (*β* = 10.41, 95% CI [5.02, 15.79]; *p* < 0.001; Cohen’s *d* = 1.21, 95% CI [0.54, 1.89]). ET-1 showed a greater decrease in the Tai Chi group (*β* = −29.04, 95% CI [−56.59, −1.48]; *p* = 0.039; Cohen’s *d* = −0.67, 95% CI [−1.31, −0.03]), as did the ET-1/NO ratio (*β* = −0.97, 95% CI [−1.61, −0.34]; *p* = 0.004; Cohen’s *d* = −0.98, 95% CI [−1.64, −0.32]). EMM trajectories were directionally consistent with these findings, showing a greater increase in NO and greater decreases in ET-1 and the ET-1/NO ratio in the Tai Chi group (Figure 7).

**Figure 6:**
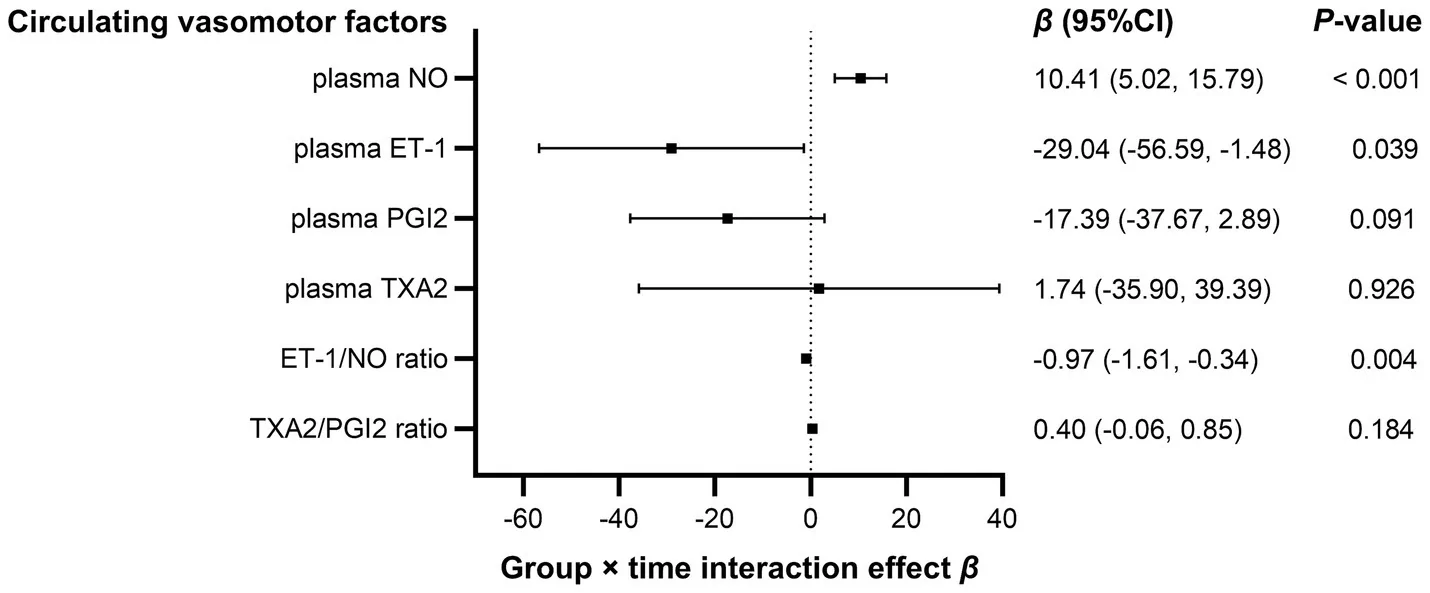
Forest plot of group-by-time interaction effects on circulating vasomotor factors. Note: Each square represents the estimated regression coefficient (*β*) for the group-by-time interaction from the linear mixed-effects model (LMM), and horizontal bars indicate the corresponding 95% confidence intervals (CIs). The vertical dashed line at *β* = 0 represents the null effect. Significant group-by-time interactions were observed for plasma NO (*p* < 0.001), ET-1 (*p* = 0.039), and the ET-1/NO ratio (*p* = 0.004), whereas no significant interactions were observed for PGI_2_, TXA_2_, or the TXA_2_/PGI_2_ ratio. Numerical estimates of *β*, 95% CIs, and corresponding *p* values are shown on the right. NO, nitric oxide; ET-1, endothelin-1; PGI_2_, prostacyclin; TXA_2_, thromboxane A_2_.

**Figure 7:**
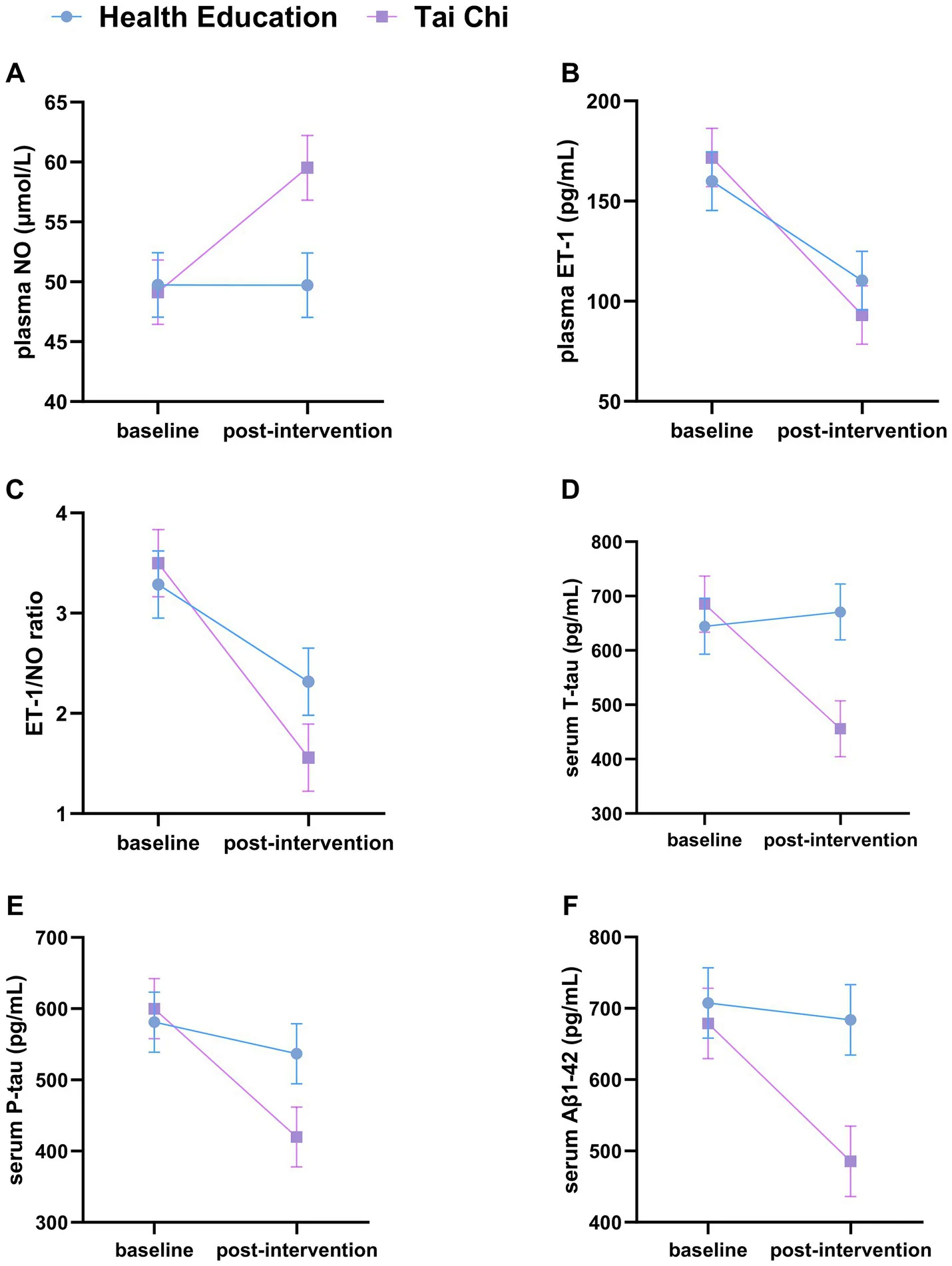
Estimated marginal mean trajectories of circulating vasomotor factors and AD-related biomarkers in the Tai Chi and health education control groups. Note: Estimated marginal means (EMMs) and 95% confidence intervals (CIs) were derived from linear mixed-effects models with group, time, and group-by-time interaction as fixed effects and participant-specific random intercepts. Subplots show changes from baseline to post-intervention in **(A)** plasma NO, **(B)** plasma ET-1, **(C)** ET-1/NO ratio, **(D)** serum T-tau, **(E)** serum P-tau, and **(F)** serum Aβ_1-42_. Blue circles indicate the health education control group, and purple squares indicate the Tai Chi group. Significant group-by-time interactions were observed for all outcomes, with *p* < 0.001 for NO, T-tau, P-tau, and Aβ_1-42_, and *p* < 0.05 for ET-1 and the ET-1/NO ratio. The direction of the between-group differences in longitudinal change varied across biomarkers, with Tai Chi showing a greater increase in NO and greater reductions in ET-1, the ET-1/NO ratio, T-tau, P-tau, and Aβ_1-42_ than the health education control group. AD, Alzheimer’s disease; NO, nitric oxide; ET-1, endothelin-1; T-tau, total tau; P-tau, phosphorylated tau; Aβ_1-42_, amyloid-beta_1–42_.

Supplementary between-group analyses of change scores corroborated the primary LMM findings (Supplementary Table S6), and individual-level distributions of changes from baseline to post-intervention in circulating vasomotor factors are presented in Supplementary Figure S3.

Baseline-adjusted ANCOVA sensitivity analyses largely aligned with the LMM findings; however, the ET-1 effect did not reach statistical significance after baseline adjustment (*p* = 0.092) (Supplementary Table S1).

#### 3.4.3 Serum Alzheimer’s disease-related biomarkers

LMMs demonstrated significant group-by-time interactions for all three circulating AD-related biomarkers (all *p* < 0.001; Table 4; Figure 8). Compared with the health education control group, the Tai Chi group exhibited greater reductions in T-tau (*β* = −255.45, 95% CI [−349.12, −161.78]; *p* < 0.001; Cohen’s *d* = −1.75, 95% CI [−2.49, −1.01]), P-tau (*β* = −135.67, 95% CI [−201.68, −69.66]; *p* < 0.001; Cohen’s *d* = −1.32, 95% CI [−2.01, −0.63]), and Aβ_1-42_ (*β* = −169.41, 95% CI [−255.73, −83.08]; *p* < 0.001; Cohen’s *d* = −1.26, 95% CI [−1.94, −0.58]). EMM trajectories showed greater reductions in all three biomarkers in the Tai Chi group than in the health education control group (Figure 7).

**Figure 8:**
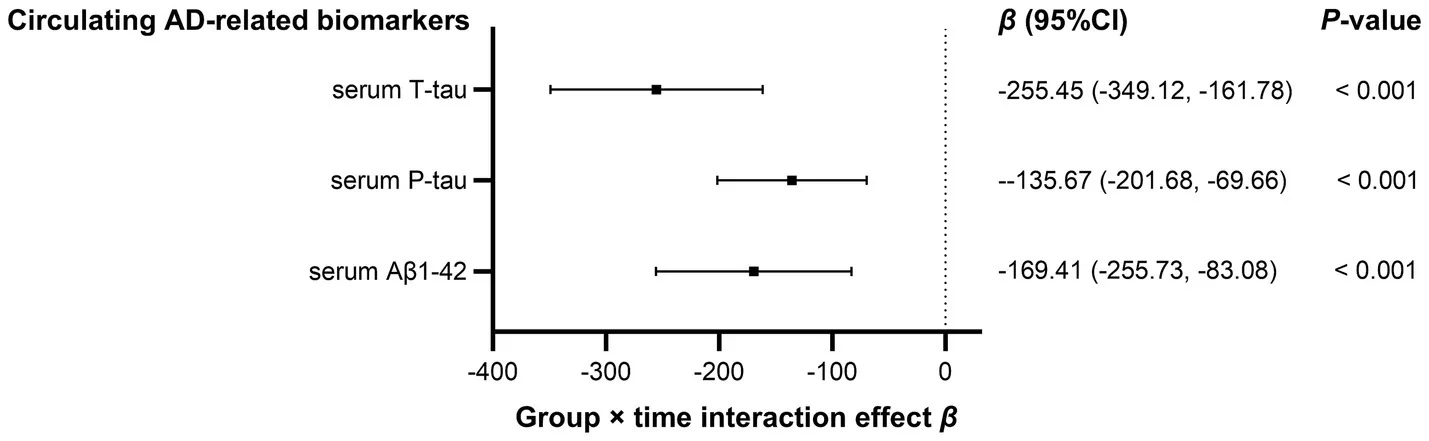
Forest plot of group-by-time interaction effects on circulating AD-related biomarkers. Note: Each square represents the estimated regression coefficient (*β*) for the group-by-time interaction from the linear mixed-effects model (LMM), and horizontal bars indicate the corresponding 95% confidence intervals (CIs). The vertical dashed line at *β* = 0 represents the null effect. All group-by-time interaction effects were statistically significant (all *p* < 0.001). Numerical estimates of β, 95% CIs, and corresponding p values are shown on the right. AD, Alzheimer’s disease; T-tau, total tau; P-tau, phosphorylated tau; Aβ_1-42_, amyloid-beta_1-42_.

Supplementary between-group analyses of change scores yielded results consistent with the primary LMM findings (Supplementary Table S6). Individual-level distributions of changes from baseline to post-intervention in circulating AD-related biomarkers are presented in Supplementary Figure S3.

Baseline-adjusted ANCOVA sensitivity analyses yielded consistent results for T-tau, P-tau, and Aβ_1-42_, with all three remaining statistically significant after baseline adjustment (all *p* < 0.001; Supplementary Table S1).

#### 3.4.4 Correlation between changes in MoCA total score and cerebrovascular and AD-related biomarkers

In exploratory analyses, improvement in MoCA total score was positively correlated with changes in global mean CBF (*r_s_* = 0.781, 95% CI [0.620, 0.879]; *p* < 0.001) and plasma NO (*r_s_* = 0.462, 95% CI [0.176, 0.676]; *p* = 0.003). Negative correlations were observed with changes in serum T-tau (*r_s_* = −0.622, 95% CI [−0.783, −0.391]; *p* < 0.001), P-tau (*r_s_* = −0.450, 95% CI [−0.667, −0.161], *p* = 0.004) and Aβ_1-42_ (*r_s_* = −0.489, 95% CI [−0.695, −0.207]; *p* = 0.001) (Table 5). No significant association was detected between changes in MoCA total score and plasma ET-1 at the Bonferroni-adjusted threshold of *p* < 0.008.

**Table 5:** Correlation between changes in MoCA total score and changes in cerebrovascular and AD-related biomarkers.

Variable	Spearman’s *r_s_*	95%CI	*P*-value
Δ Global mean CBF	0.781	(0.620, 0.879)	**< 0.001**
Δ NO	0.462	(0.176, 0.676)	**0.003**
Δ ET-1	−0.302	(−0.557, 0.003)	0.058
Δ T-tau	−0.622	(−0.783, −0.391)	**< 0.001**
Δ P-tau	−0.450	(−0.667, −0.161)	**0.004**
Δ Aβ_1-42_	−0.489	(−0.695, −0.207)	**0.001**

Changes were calculated as post-intervention values minus baseline values. r_s_ represents Spearman’s rank correlation coefficient. p-values were adjusted using the Bonferroni correction for six comparisons, with a corrected significance threshold of *p* < 0.008.

MoCA, Montreal Cognitive Assessment; CI, confidence interval; CBF, cerebral blood flow; NO, nitric oxide; ET-1, endothelin-1; T-tau, total tau; P-tau, phosphorylated tau; Aβ_1-42_, amyloid-β_1-42_.

### 3.5 Safety outcomes

The mean attendance rate in the Tai Chi group was 92.5%, with all participants meeting the prespecified adherence threshold of ≥ 80%. No intervention-related adverse events were reported in either group. All reported adverse events were mild (Grade 1) according to CTCAE version 5.0, with no moderate or severe adverse events observed. One case of transient blood pressure elevation in the health education control group and one case of mild knee discomfort in the Tai Chi group (occurring on day 3 and resolving spontaneously within 24 h with rest, without recurrence) were both judged unrelated to the intervention. The incidence of non-intervention-related adverse events was low and comparable between groups (5.0% vs. 5.0%).

## 4 Discussion

In this single-center, parallel-group randomized controlled trial, a 12-week program of standardized 24-form Tai Chi was associated with improvements in cognitive function in postmenopausal women with MCI. Improvements were observed not only in global cognition but also in domains particularly vulnerable in early cognitive decline, including delayed recall, visuospatial/executive function, and attention. These effects were consistent with our *a priori* hypothesis and were accompanied by convergent changes in cerebrovascular outcomes, including increased resting CBF in cognition-related regions, a more favorable vasomotor profile (increased NO, reduced ET-1, and a lower ET-1/NO ratio), and reductions in circulating AD-related biomarkers (T-tau, P-tau, and Aβ_1-42_). Resting CBF and circulating vascular biomarkers are informative indicators of cerebrovascular health. The coordinated improvements observed in both cerebral perfusion and vasomotor regulation suggest that Tai Chi may favorably influence cerebrovascular function and thereby support the metabolic demands of neural tissue. However, because NVC was not directly assessed using task-based or other dynamic neuroimaging paradigms, these findings should not be interpreted as direct evidence of altered NVC. Rather, they may reflect favorable changes in cerebral perfusion and vascular regulation that could contribute to neurovascular regulation.

The observed changes in resting CBF were larger than those reported in many previous exercise studies; however, direct quantitative comparisons are limited because prior meta-analyses have primarily evaluated transcranial Doppler-derived cerebral blood velocity rather than ASL-derived tissue CBF (Prudente et al., 2023; Li W. et al., 2025a; Li X. et al., 2025b). Importantly, ROI measurement reproducibility was good (CV, 3.2–5.7%), suggesting that variability attributable to repeated ROI delineation was substantially smaller than the observed between-group differences. However, these estimates do not constitute a test–retest assessment of the full ASL acquisition and quantification process, and therefore cannot establish temporal stability of CBF measurements across separate scanning sessions. Nevertheless, ASL-derived resting CBF is influenced by multiple physiological and methodological factors, including arterial transit time, vascular CO_2_ reactivity, systemic hemodynamics status, hydration, and acquisition-related variability; therefore, these findings should be interpreted cautiously.

Several factors may have contributed to the relatively large between-group difference. First, the Tai Chi group had relatively low baseline global CBF (49.84 mL·100 g^−1^·min^−1^), potentially allowing greater capacity for improvement. Second, the health education control group exhibited an approximately 10% decline in global CBF over 12 weeks, which contributed to the observed group contrast; this pattern is broadly consistent with reports of age-related reductions in cerebral perfusion among older adults (Guo et al., 2024a), although the mechanisms underlying this short-term decline remain uncertain. Third, the present study focuses on postmenopausal women with MCI, a population characterized by increased cerebrovascular vulnerability and potentially greater responsiveness to interventions targeting vascular function. However, differences in participant characteristics, intervention protocols, MRI acquisition parameters, ASL processing pipelines, and CBF quantification approaches limit direct comparisons with previous studies. Given the exploratory design, small sample size, and absence of a time- and attention-matched active comparator, the magnitude of the observed CBF effects should not be interpreted as definitive evidence of clinical efficacy. Rather, these findings are hypothesis-generating and warrant confirmation in larger studies using standardized ASL protocols and independent test–retest assessments.

These findings are particularly relevant given the increased susceptibility of postmenopausal women to cognitive decline, a risk that may be associated with the loss of endogenous estrogen following ovarian senescence (Sochocka et al., 2023). Estrogen plays a central role in maintaining NVU integrity through multiple interconnected pathways. Beyond regulating eNOS and preserving the balance between NO and ET-1 (Lv et al., 2025), estrogen exerts anti-inflammatory effects, supports mitochondrial function, and helps maintain blood–brain barrier (BBB) integrity (Heo et al., 2023; Collignon et al., 2024; Hu et al., 2024).

The loss of this multi-system protection after menopause contributes to endothelial dysfunction, chronic neuroinflammation, reduced cerebral perfusion, BBB disruption, and accelerated AD pathology (Fréneau et al., 2022; Wang et al., 2022; Guo et al., 2024a). Accumulating evidence suggests that mind–body exercises such as Tai Chi may confer cognitive benefits through these same pathways: reducing systemic and central inflammation (via downregulation of pro-inflammatory cytokines), enhancing mitochondrial biogenesis and function in neurons and endothelial cells, and preserving BBB integrity through upregulation of tight junction proteins (Wang et al., 2022). Despite this well-established biology, most exercise trials in MCI have included mixed-sex populations and have not specifically addressed these sex-specific mechanisms. Our study provides targeted evidence in postmenopausal women, showing that Tai Chi was associated with increased NO levels and a reduced ET-1/NO ratio, consistent with improved endothelial function. The observed association between changes in NO and cognitive performance further supports vasomotor regulation as a key pathway linking the intervention to cognitive benefit. Future studies should delineate the relative contributions of inflammation, mitochondrial function, and BBB integrity to Tai Chi-induced cognitive improvement in this high-risk population.

Although both groups showed reductions in vasoconstrictive markers (ET-1 and TXA_2_), only the Tai Chi group demonstrated meaningful cognitive improvement. Several factors may explain this divergence. First, only Tai Chi led to a significant increase in NO, a critical mediator of activity-dependent vasodilation; reductions in vasoconstrictors alone are unlikely to improve vascular responsiveness and cerebral perfusion in the absence of sufficient NO signaling. Second, these differences were reflected in cerebral perfusion: Tai Chi increased CBF across all examined regions, whereas the control group showed a decline over the 12-week period, consistent with expected age-related trends. Third, Tai Chi integrates coordinated movement, controlled breathing, and focused attention, which may provide additional neurocognitive benefits beyond vascular effects alone. This multimodal nature may help explain why health education, despite modest improvements in some vascular markers, did not translate into measurable cognitive gains.

Notably, most prior Tai Chi trials in MCI have focused primarily on cognitive outcomes, with relatively few incorporating biomarker assessments. This gap highlights the contribution of our study, which provides preliminary evidence of coordinated changes in cerebral perfusion, vascular function and circulating AD-related biomarkers following the Tai Chi intervention. The dissociation observed in the control group, characterized by partial improvement in vascular markers without corresponding cognitive benefits, further supports a multimodal mechanism of action. Specifically, the cognitive benefits of Tai Chi likely arise from the synergistic effects of enhanced vascular function and direct neuroplastic changes that are not fully captured by peripheral blood markers. This may help explain why health education, despite modest improvements in some vascular markers, did not translate into measurable cognitive improvement.

The observed increases in CBF across cognition-related brain regions are likely to have direct functional implications for the cognitive improvements seen in this study. The bilateral middle frontal gyrus, anterior cingulate cortex, hippocampus, and parahippocampal gyrus are key components of the default mode and executive control networks and are particularly vulnerable to estrogen deficiency and early AD pathology (Ramli et al., 2023; Losinski et al., 2024). Importantly, these same regions have been shown to undergo neuroplastic changes in response to Tai Chi. A 2023 systematic review by Jasim et al. reported that Tai Chi is associated with increased neuronal activity, enhanced functional connectivity within the default mode and executive control networks, and preservation of gray matter volume in these regions (Jasim et al., 2023). Our findings extend this work by suggesting that improved regional cerebral perfusion may represent one potential mechanism associated with these structural and functional neuroplastic adaptations. Furthermore, regional perfusion changes showed domain-specific correspondence with cognitive gains. Increased perfusion in the hippocampus and parahippocampal gyrus aligned with improvements in delayed recall (Guo et al., 2024b), whereas enhanced CBF in the middle frontal gyrus and anterior cingulate cortex paralleled gains in visuospatial/executive function and attention (Zhang et al., 2021a). These findings suggest that the cognitive benefits of Tai Chi may be mediated, at least in part, by targeted improvements in regional cerebral perfusion, supporting more effective perfusion-metabolism coupling. The positive correlation between increases in global mean CBF and improvements in MoCA scores further supports this interpretation.

The concurrent reductions in circulating T-tau, P-tau, and Aβ_1-42_ observed in the Tai Chi group may point to several plausible, non-mutually exclusive mechanisms, although causal relationships cannot be established. First, favorable changes in vasomotor regulation and nitric oxide bioavailability may contribute to improved cerebrovascular function and cerebral perfusion, potentially supporting neural metabolic demands. Second, enhanced cerebral blood flow could theoretically support neuronal energy supply and metabolic stability, which may influence neuronal homeostasis and potentially affect circulating tau dynamics (Houck et al., 2025). Third, systemic effects of Tai Chi, including changes in vascular and metabolic function, may influence peripheral biomarker dynamics independently of central pathology. The observed inverse association between changes in P-tau and cognitive performance provides additional clinical context but does not establish a causal or mechanistic relationship. Importantly, circulating T-tau, P-tau, and Aβ₁-42 are indirect biomarkers and cannot directly reflect brain pathology; therefore, reductions in these circulating biomarkers should not be interpreted as evidence of altered AD pathology or enhanced clearance, as peripheral concentrations may reflect multiple biological processes. These findings require confirmation using central biomarkers such as CSF measures or amyloid imaging. Although cerebrovascular regulation has been hypothesized to interact with glymphatic transport, glymphatic function and sleep-related measures were not assessed in this study; therefore, this interpretation remains speculative and requires confirmation in future studies using direct glymphatic assessments.

In addition, our findings are consistent with, and extend, recent evidence supporting Tai Chi for cognitive improvement in individuals with MCI. A 2024 meta-analysis of nine RCTs (*n* = 1,046) reported significant improvements in global cognition and delayed recall with Tai Chi compared with health education, stretching, and brisk walking (Shao et al., 2024). Subgroup analyses further indicated that the 24-form Tai Chi protocol was associated with the greatest gains in global cognitive function. A 2025 dose–response meta-analysis of 17 RCTs (*n* = 2,227) further identified three sessions per week, 45–60 min per session, over 12 weeks as a regimen associated with the greatest cognitive benefit (Wen et al., 2025), closely matching our protocol. Consistent findings were also reported in a large 2023 randomized clinical trial of adults with type 2 diabetes and MCI (*n* = 328), in which 24-form Tai Chi (three sessions per week, 60 min per session, for 24 weeks) improved MoCA scores compared with brisk walking and health education controls, with benefits sustained at 36-week follow-up (Chen et al., 2023). Nevertheless, the magnitude of the cognitive effect in our study was substantially larger than that reported previously; a recent meta-analysis estimated a pooled SMD of 0.526 for global cognition (Wang and Wang, 2025), compared with a Cohen’s *d* of 3.28 for MoCA total score in our study. This discrepancy warrants caution and may partly reflect the small sample size, sampling variability, expectation and other non-specific intervention effects, as well as the absence of a time-, attention-, and activity-matched active comparator. Prior trials have largely focused on cognitive outcomes, with limited characterization of cerebral perfusion, vascular function, and circulating AD-related biomarkers, particularly in postmenopausal women with MCI. Thus, our findings provide preliminary, hypothesis-generating evidence of coordinated cognitive and cerebrovascular-related changes following Tai Chi.

While health education produced modest improvements in systemic vasomotor balance, only Tai Chi led to a significant increase in circulating NO. This distinction is mechanistically important, as NO is the primary mediator of endothelium-dependent vasodilation and a key regulator of vascular tone. The selective increase in NO bioavailability in the Tai Chi group provides a plausible physiological explanation for the observed improvements in cerebral perfusion and the associated cognitive benefits, which were not evident in the control group.

Several outcomes did not differ significantly between groups, including the abstraction and orientation domains of the MoCA and the prostaglandin-related markers (PGI_2_, TXA_2_, and the TXA_2_/PGI_2_ ratio). The lack of improvement in higher-order cognitive domains may reflect the relatively short duration of the intervention, as changes in these domains often require longer exposure. The similar changes in prostaglandin-related markers across groups may be attributable to shared dietary and lifestyle recommendations included in the health education program, which are known to influence prostaglandin metabolism.

From a clinical perspective, these findings address an important gap in strategies for early AD prevention. Postmenopausal women with MCI have limited access to safe and effective interventions, as current disease-modifying therapies are largely restricted to early-stage AD and are associated with high costs and potential adverse effects (Hlávka et al., 2022). Tai Chi is an accessible and well-tolerated mind–body exercise that does not require specialized equipment. In this study, it produced meaningful cognitive improvements alongside favorable vascular changes, suggesting a mechanism relevant to estrogen deficiency-related vascular vulnerability (Cai et al., 2025). Given its feasibility and accessibility, Tai Chi may represent a practical approach for community- and primary care-based interventions aimed at reducing dementia risk in postmenopausal women.

Taken together, our findings support a multilevel vascular model through which 12 weeks of Tai Chi may improve cognitive function in postmenopausal women with MCI (Figure 9). Tai Chi appears to first modulate systemic vasomotor balance, as reflected by increased nitric oxide availability and a reduced ET-1/NO ratio. These changes are consistent with improved endothelial function and may contribute to enhanced global and regional cerebral blood flow in key cognitive regions, including the hippocampus and anterior cingulate cortex. Improved perfusion may, in turn, support neuronal energy metabolism and reduce neuronal stress, potentially influencing the release and peripheral metabolism of tau and Aβ proteins. Although circulating T-tau, P-tau, and Aβ_1-42_ are indirect indicators of central pathology, their observed reductions are consistent with changes in peripheral biomarker dynamics. Collectively, these changes are associated with meaningful improvements in global cognition, episodic memory, and executive function. This framework provides a coherent mechanistic basis for the cognitive effects of Tai Chi and highlights the potential of mind–body interventions as non-pharmacological strategies for early AD prevention. Future studies incorporating direct measures of cerebral metabolism and CSF biomarkers are warranted to further validate these mechanistic pathways.

**Figure 9:**
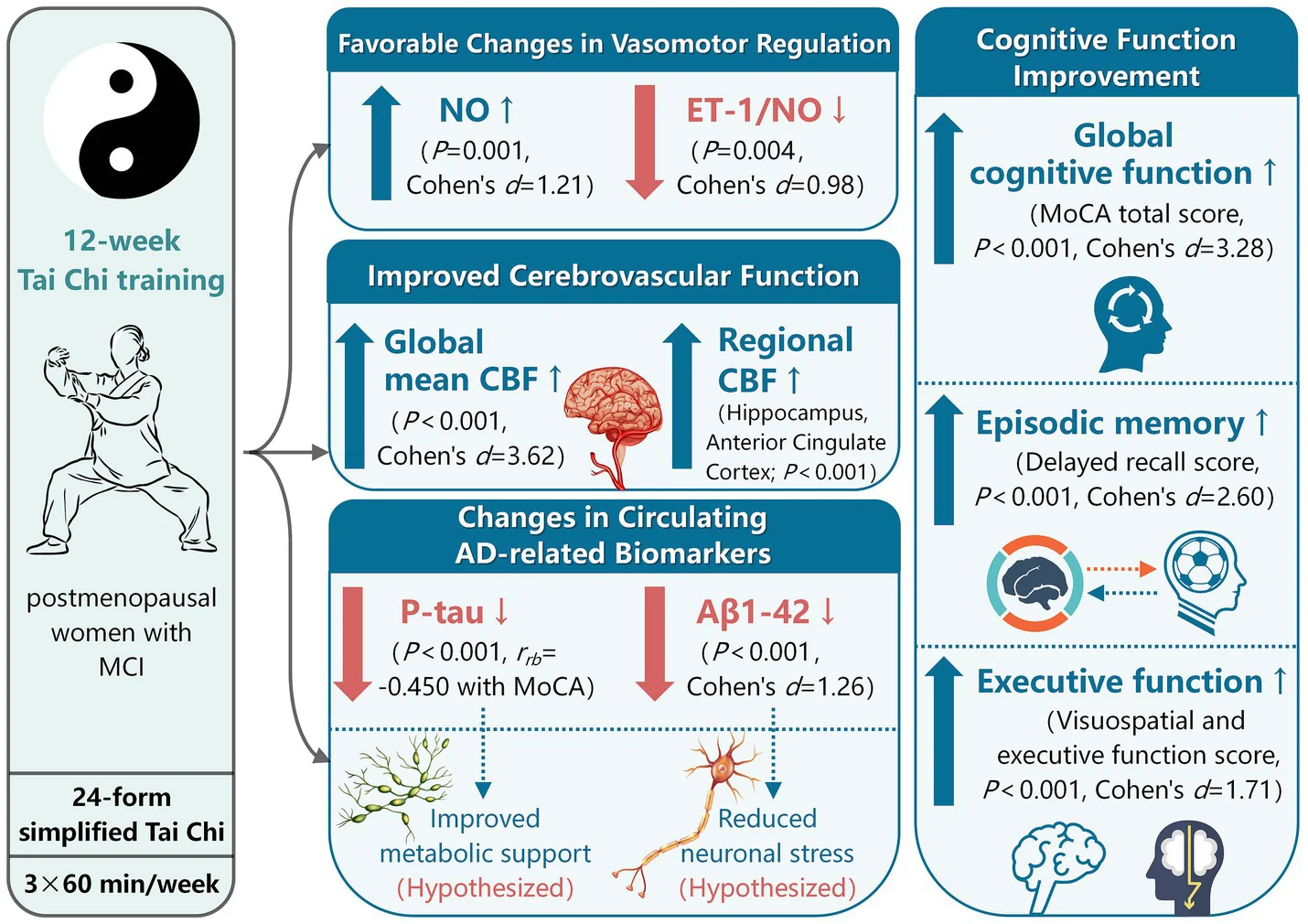
Conceptual schematic of observed changes in cognitive function, cerebral perfusion, vasomotor regulation, and circulating AD-related biomarkers following Tai Chi in postmenopausal women with MCI. Note: Solid arrows indicate observed changes; dashed arrows indicate hypothesized relationship or mechanisms that were not directly assessed. NO, nitric oxide; ET-1, endothelin-1; CBF, cerebral blood flow; P-tau, phosphorylated tau; Aβ_1-42_, amyloid-beta_1_-42; MoCA, Montreal Cognitive Assessment; *rrb*, rank-biserial correlation coefficient; MCI, mild cognitive impairment.

Several limitations should be considered. First, this was a small, single-center study involving a relatively homogeneous cohort of postmenopausal women from Fujian Province, China. While this may have reduced variability and enhanced internal validity, it may also limit generalizability to broader populations. The small sample size limits the precision of the effect estimates and may increase the risk of inflated effect estimates. The relatively large effects observed for MoCA total score and global mean CBF may therefore partly reflect sampling variability and other non-specific intervention effects. It should also be noted that the reproducibility analyses reported here assessed the within-dataset repeatability and inter-rater reproducibility of ROI-based CBF measurements, rather than the test–retest stability of CBF measurements obtained from repeated MRI acquisitions at different time points. Thus, although these findings support the consistency of the ROI-based CBF measurement procedure, they do not establish the temporal stability of CBF measurements or exclude physiological or methodological sources of temporal variation. In addition, the sample size calculation was partly informed by unpublished pilot data, which may limit the reproducibility of the assumptions underlying the sample size estimation. In addition, the observed changes in cerebral perfusion and circulating AD-related biomarkers should not be interpreted as definitive evidence of specific neurovascular mechanisms or underlying Alzheimer’s disease pathology. Therefore, these findings should be regarded as preliminary and hypothesis-generating rather than confirmatory. Second, the 12-week intervention and two assessment time points preclude conclusions about the durability of the observed effects or their impact on long-term outcomes such as progression to AD. Longer follow-up with repeated assessments would help clarify the temporal dynamics of these changes. Third, AD-related biomarkers were measured in serum rather than cerebrospinal fluid or via amyloid imaging; as such, they represent indirect markers that may be influenced by peripheral processes and do not directly reflect central pathology. Fourth, Aβ_1-40_ was not measured, precluding calculation of the Aβ_1-42_/Aβ_1-40_ ratio. Because this ratio provides more informative evidence regarding amyloid pathology than Aβ_1-42_ alone, the observed changes in circulating Aβ_1-42_ should be interpreted cautiously and should not be considered definitive evidence of altered amyloid pathology. Fifth, our sample was not specifically classified as AD-related or vascular MCI because disease-specific biomarkers or imaging measures, such as CSF biomarkers and amyloid PET, were not obtained. Thus, participants may have included heterogeneous etiologies, and whether the effects of Tai Chi differ across MCI subtypes warrants further investigation. Sixth, daily physical activity was assessed by self-report rather than objective measures, which may introduce reporting bias. Seventh, the health education control group cannot disentangle Tai Chi-specific effects from general physical activity or non-specific factors such as attention, social interaction, expectations of benefit, or physical activity, as it lacked a structured physical activity and had substantially less contact time, supervision, and social interaction. Therefore, some of the observed between-group differences may reflect these non-specific effects rather than Tai Chi-specific effects, warranting cautious interpretation of our findings as preliminary evidence of potential benefits associated with a structured Tai Chi intervention compared with health education alone, rather than definitive proof of Tai Chi-specific efficacy. Future trials incorporating active exercise comparators are needed to isolate Tai Chi’s unique contribution. Eighth, blinding of participants was not feasible given the nature of the Tai Chi intervention. Although outcome assessors, MRI readers, laboratory personnel, and the statistician were masked to group allocation, participants’ awareness of their assigned treatment could have introduced non-specific effects related to treatment expectancy, motivation and engagement. This limitation is particularly relevant to the cognitive outcomes, because the MoCA is a brief cognitive screening instrument rather than a comprehensive neuropsychological battery and may not capture the breadth and depth of cognitive change across individual domains. Accordingly, the MoCA total score and domain-specific scores should be interpreted as screening-level measures of overall cognitive performance and performance within selected cognitive domains, respectively. In addition, the same MoCA version was administered at baseline and post-intervention without an alternate or parallel form, which may have introduced practice, training, and rehearsal effects, particularly for verbal recall and visuospatial/executive tasks. Although the 12-week interval between assessments may have reduced the extent of such effects, they cannot be fully excluded. Therefore, the observed cognitive improvements should be interpreted cautiously, as the extent to which they reflect intervention-related cognitive change versus nonspecific practice or familiarity effects cannot be determined from the present design. Future studies should consider using alternate or parallel cognitive assessment forms and, where feasible, comprehensive neuropsychological batteries to provide a broader and more robust characterization of cognitive change.

## 5 Conclusion

This exploratory randomized controlled trial provides preliminary evidence that a 12-week program of standardized 24-form Tai Chi was associated with improvements in overall cognitive performance as assessed by the MoCA and in performance on selected MoCA cognitive domains in postmenopausal women with MCI. These cognitive changes were accompanied by increased cerebral perfusion, a more favorable vasomotor profile, and reductions in circulating AD-related biomarkers. While these findings are encouraging, they should be considered preliminary and hypothesis-generating. Larger, multicenter studies with longer follow-up are warranted to determine the reproducibility and durability of these findings and their relevance to long-term clinical outcomes.

## Data Availability

The raw data supporting the conclusions of this article will be made available by the authors, without undue reservation.
