# Supplementary material for: Effects of 12-week 24-form Tai Chi on cognitive function, cerebral perfusion, and vascular function in postmenopausal women with mild cognitive impairment: an exploratory randomized controlled trial
Source: Front Aging Neurosci. 2026 Sep 25;18:1880915. doi: 10.3389/fnagi.2026.1880915 (PMC13649657; doi:10.3389/fnagi.2026.1880915)
Supplement: Supplementary file 1 [file Data_Sheet_1.doc]

**Supplementary Materials**

**Supplementary Method**

**Supplementary Method S1** Detailed cerebral blood flow measurement protocol.

**Supplementary Tables**

**Supplementary Table S1** Baseline-Adjusted ANCOVA Comparisons Between Groups at Post-Intervention

**Supplementary Table S2** Sensitivity Analysis Using Baseline-Adjusted Linear Models With Group × Baseline Interactions

**Supplementary Table S3** Simple Slopes Analysis of Between-Group Differences in Post-Intervention Outcomes at Selected Baseline Levels

**Supplementary Table S4** Changes in the MoCA Total and Domain-specific Scores at Baseline and Post-Intervention.

## Supplementary Table S5 Changes in CBF of cognition-related brain regions at Baseline and Post-Intervention.

**Supplementary Table S6** Changes in Circulating Vasomotor Factors and Serum AD-related Biomarkers at Baseline and Post-Intervention.

Groups at Post-Intervention.

**Supplementary Figures**

**Supplementary Figure S1** Individual-level distributions of changes from baseline to post-intervention in MoCA total score and three domain-specific scores.

## Supplementary Figure S2 Individual-level distributions of changes from baseline to post-intervention in global mean CBF and CBF of cognition-related brain regions.

## Supplementary Figure S3 Individual-level distributions of changes from baseline to post-intervention in circulating vasomotor and serum AD-related biomarkers.

**Supplementary Method**

**Supplementary Method S1** Detailed cerebral blood flow assessment protocol.

**S1.1 MRI acquisition**

Regional and global cerebral blood flow (CBF) measurements were obtained using pseudo-continuous arterial spin labeling (pCASL) on a 3.0T MRI scanner (Signa HDX, GE Healthcare, Milwaukee, WI, USA).

The pCASL acquisition parameters were as follows: labeling duration, 1450 ms; post-labeling delay (PLD), 1525 ms; repetition time (TR), 4368 ms; echo time (TE), 10.5 ms; slice thickness, 4 mm; interslice gap, 1 mm; field of view (FOV), 24 × 24 cm; acquisition matrix, 128 × 128; voxel size, 1.88 × 1.88 × 4.00 mm^3^; and 30 label-control pairs. Thirty axial slices were acquired to achieve whole-brain coverage, with a total acquisition time of approximately 2.2 minutes.

**S1.2 Region-of-interest definition and CBF quantification**

Predefined cognition-related regions were selected based on their established involvement in cognitive impairment, including the bilateral middle frontal gyrus, anterior cingulate cortex, hippocampus, and parahippocampal gyrus. Whole-brain mean CBF was calculated from the whole-brain perfusion map.

ROIs were manually delineated on standardized axial slices aligned to the anterior commissure–posterior commissure (AC-PC) line. Each ROI was approximately 50-100 mm^2^, depending on anatomical characteristics, and was restricted to gray matter while avoiding sulci, large vessels, cerebrospinal fluid, and visible imaging artifacts.

Two board-certified radiologists, blinded to group allocation, independently performed ROI delineation. Each ROI was measured in triplicate, and the average value was used for statistical analysis. For bilateral structures, the mean value of both hemispheres was calculated.

**S1.3 Image quality control**

All ASL datasets were visually inspected before quantitative analysis for excessive head motion, incomplete brain coverage, labeling-related artifacts, susceptibility artifacts, and other image-quality issues. Images with inadequate coverage or substantial artifacts affecting predefined ROIs were excluded from quantitative assessment. The same acquisition parameters and quantitative assessment procedures were applied consistently at baseline and post-intervention assessments.

**S1.4 Measurement reproducibility**

To evaluate the repeatability and inter-rater reproducibility of ROI-based CBF measurements, intra-rater and inter-rater reliability were assessed using intraclass correlation coefficients (ICCs). Intra-rater reliability was evaluated using repeated measurements performed by the same radiologist on the same ASL dataset, whereas inter-rater reliability was assessed by comparing measurements obtained independently by the two radiologists. Each ROI was measured in triplicate, and the average value was used for statistical analysis. The coefficient of variation (CV) was calculated as the standard deviation divided by the mean of repeated measurements and expressed as a percentage to quantify measurement variability. Intra-rater ICCs ranged from 0.89 to 0.94, inter-rater ICCs ranged from 0.85 to 0.91, and CVs ranged from 3.2% to 5.7%, indicating good within-dataset repeatability and inter-rater reproducibility of ROI-based CBF measurements. These analyses did not assess test-retest stability across repeated MRI acquisitions performed at different time points.

**Supplementary Tables**

**Supplementary Table S1** Baseline-Adjusted ANCOVA Comparisons Between Groups at Post-Intervention.

| Outcome | Health Education control Group (n = 20),  adjusted mean ± SE | Tai Chi Group   (n = 20),  adjusted mean ± SE | Adjusted between-group difference (95% CI) | *F* (*df*₁*, df*₂) | *P*-value | Partial *η*² |
| --- | --- | --- | --- | --- | --- | --- |
| NO (μmol/L) | 49.73 ± 1.54 | 59.52 ± 1.54 | 9.79 (5.38, 14.19) | 20.26 (1, 37) | **< 0.001** | 0.354 |
| ET-1 (pg/mL) | 111.22 ± 7.69 | 92.25 ± 7.69 | -18.97 (-41.19, 3.26) | 2.99 (1, 37) | 0.092 | 0.075 |
| PGI_2_ (pg/mL) | 191.69 ± 7.13 | 176.37 ± 7.13 | -15.33 (-35.95, 5.30) | 2.27 (1, 37) | 0.141 | 0.058 |
| TXA_2_ (pg/mL) | 182.83 ± 10.07 | 170.11 ± 10.07 | -12.72 (-41.82, 16.37) | 0.79 (1, 37) | 0.381 | 0.021 |
| ET-1/NO | 2.33 ± 1.17 | 1.54 ± 1.17 | -0.79 (-1.28, -0.29) | 10.49 (1, 37) | **0.003** | 0.221 |
| TXA_2_/PGI_2_ | 0.96 ± 0.08 | 1.04 ± 0.08 | 0.08 (-0.14, 0.30) | 0.52 (1, 37) | 0.478 | 0.014 |
| T-tau (pg/mL) | 674.21 ± 23.58 | 452.51 ± 23.58 | -221.69 (-289.73, -153.66) | 43.59 (1, 37) | **< 0.001** | 0.541 |
| P-tau (pg/mL) | 540.70 ± 20.10 | 415.91 ± 20.10 | -124.80 (-182.54, -67.06) | 19.18 (1, 37) | **< 0.001** | 0.341 |
| Aβ_1_-42 (pg/mL) | 680.22 ± 23.67 | 489.13 ± 23.67 | -191.10 (-259.21, -122.98) | 32.32 (1, 37) | **< 0.001** | 0.466 |

Note: Data are presented as estimated marginal means ± SE. Each ANCOVA model was adjusted for its corresponding baseline outcome value as the sole covariate. The adjusted between-group difference was calculated as the Tai Chi group minus the health education control group; positive values indicate higher post-intervention outcome values in the Tai‑Chi group. Only outcomes satisfying the assumptions of normally distributed residuals, homogeneity of variance, and homogeneity of regression slopes were included. The MoCA total score and global cerebral blood flow were not analyzed using ANCOVA because the assumption of homogeneity of regression slopes was violated.

**Supplementary Table S2** Sensitivity Analysis Using Baseline-Adjusted Linear Models With Group × Baseline Interactions

| Parameter | B (95% CI) | SE | *t* | *P*-value |
| --- | --- | --- | --- | --- |
| MoCA total score model (Adjusted *R*^2^ = 0.846) | | | | |
| Intercept | 28.00 (27.37, 28.63) | 0.31 | 90.43 | **< 0.001** |
| Group (Tai Chi vs Health Education) | 4.92 (4.03, 5.81) | 0.44 | 11.23 | **< 0.001** |
| Baseline MoCA (centered) | 0.96 (0.56, 1.36) | 0.20 | 4.90 | **< 0.001** |
| Group × Baseline interaction | -0.69 (-1.28, -0.10) | 0.29 | -2.38 | **0.023** |
| Global mean CBF model (Adjusted *R^2^* = 0.793) |  |  |  |  |
| Group (Tai Chi vs Health Education) | 14.43 (12.02, 16.85) | 1.19 | 12.12 | **< 0.001** |
| Baseline CBF (centered) | 0.86 (0.42, 1.30) | 0.22 | 3.95 | **< 0.001** |
| Group × Baseline interaction | 0.70 (0.06, 1.33) | 0.31 | 2.23 | **0.032** |

Note: Data are presented as regression coefficient (B) with 95% confidence intervals (CIs), standard errors (SEs), *t* statistics, and *P* values. The health education group was set as the reference category. Baseline values were grand-mean centered. The post-intervention outcome was modeled as a function of treatment group, \centered baseline value, and the group × baseline interaction. The group coefficient represents the estimated between-group difference in the post-intervention outcome at the mean baseline level. A significant group × baseline interaction indicates that the magnitude of the between-group difference at follow-up varies according to baseline status.

Abbreviations: CI, confidence interval; SE, standard error; MoCA: Montreal Cognitive Assessment; CBF: cerebral blood flow.

**Supplementary Table S3** Simple Slopes Analysis of Between-Group Differences in Post-Intervention Outcomes at Selected Baseline Levels

| Baseline Level | Adjusted Mean Difference (95% CI) | *P*-value |
| --- | --- | --- |
| MoCA total score (points) | | |
| 25th percentile (23) | 5.58 (4.53, 6.62) | **< 0.001** |
| Median (23.95) | 4.92 (4.03, 5.81) | **< 0.001** |
| 75th percentile (25) | 4.19 (3.11, 5.28) | **< 0.001** |
| Global mean CBF (mL·100 g^-1^·min^-1^) |  |  |
| 25th percentile (48.42) | 12.84 (10.01, 15.67) | **< 0.001** |
| Median (50.71) | 14.43 (12.02, 16.85) | **< 0.001** |
| 75th percentile (53.93) | 16.68 (13.53, 19.83) | **< 0.001** |

Note: Adjusted mean differences (Tai Chi group minus health education control group) were estimated from baseline-adjusted linear models including the group × baseline interaction, evaluated at the 25th percentile, median, and 75th percentile of the respective baseline distribution. Positive values indicate higher post-intervention values in the Tai Chi group. For MoCA total score, the between-group difference was greater at lower baseline MoCA scores; for global mean CBF, the between-group difference increased with higher baseline CBF.

Abbreviations: CI, confidence interval; MoCA: Montreal Cognitive Assessment; CBF, cerebral blood flow.

**Supplementary Table S4** Changes in the MoCA Total and Domain-specific Scores at Baseline and Post-Intervention.

| Cognitive Domain | Health Education control Group (n = 20) | | | | Tai Chi Group (n = 20) | | | | Effect Size  *r_rb_* [95%CI] | Between-group *P*-value |
| --- | --- | --- | --- | --- | --- | --- | --- | --- | --- | --- |
|  | Pre | Post | d | within-group *P*-value | Pre | Post | d | within-group *P*-value |  |  |
| Total MoCA Score | 25.00(23.00, 25.00) | 24.50(20.00, 25.75) | 0.00(-2.50, 0.75) | 0.098 | 25.00(24.00, 25.00) | 28.00(27.00, 29.00) | 4.00(3.00, 5.00) | **< 0.001** | 0.87 [0.76, 0.93] | **< 0.001** |
| Visuospatial/Executive Function | 3.50(3.00, 4.00) | 3.00(3.00, 4.00) | 0.00(-0.75, 0.00) | 0.480 | 4.00(3.00, 4.00) | 5.00(5.00, 5.00) | 1.00(1.00, 1.00) | **< 0.001** | 0.69 [0.49, 0.83] | **< 0.001** |
| Naming | 3.00(3.00, 3.00) | 3.00(2.00, 3.00) | 0.00(0.00, 0.00) | **0.035** | 3.00(3.00, 3.00) | 3.00(3.00, 3.00) | 0.00(0.00, 0.75) | 0.157 | 0.33 [0.02, 0.58] | 0.035 |
| Attention | 5.00(4.25, 5.00) | 5.00 (4.00, 6.00) | 0.00 (0.00, 0.00) | 0.557 | 5.00(5.00, 5.00) | 6.00(6.00, 6.00) | 1.00(1.00, 1.00) | **< 0.001** | 0.65 [0.43, 0.80] | **< 0.001** |
| Language | 2.00(1.25, 3.00) | 2.50(1.00, 3.00) | 0.00 (0.00, 0.00) | 0.414 | 2.00(1.25, 3.00) | 3.00(2.00, 3.00) | 0.00(0.00, 0.75) | **0.034** | 0.33 [0.02, 0.58] | 0.035 |
| Abstraction | 1.00(1.00, 2.00) | 1.00(1.00, 2.00) | 0.00 (0.00, 0.00) | 0.655 | 2.00(1.00, 2.00) | 2.00(2.00, 2.00) | 0.00(0.00, 1.00) | 0.157 | 0.22 [-0.10, 0.50] | 0.159 |
| Delayed Recall | 3.00(3.00, 4.00) | 3.00(3.00, 4.00) | 0.00 (0.00, 0.00) | 0.414 | 3.00(3.00, 3.00) | 5.00(4.00, 5.00) | 1.00(1.00, 2.00) | **< 0.001** | 0.81 [0.67, 0.90] | **< 0.001** |
| Orientation | 6.00(6.00, 6.00) | 6.00(5.25, 6.00) | 0.00 (0.00, 0.00) | 0.414 | 6.00(6.00, 6.00) | 6.00(6.00, 6.00) | 0.00(0.00, 0.00) | 0.157 | 0.22 [-0.10, 0.50] | 0.163 |

Note: Data are presented as median (P_25_, P_75_). d = change score (post-intervention minus baseline). *r_rb_* = rank-biserial correlation coefficient, calculated as *r_rb_* = |Z|/$\sqrt{N}$ (N = 40). 95% CIs for *r_rb_* were derived via Fisher *Z*-transformation. Effect sizes: 0.1 = small, 0.3 = medium, 0.5 = large, ≥ 0.8 = very large. The MoCA total score was considered the primary outcome (α = 0.05). Bonferroni correction was applied for the seven exploratory MoCA subdomain analyses (*P* < 0.007).

Abbreviations: MoCA, Montreal Cognitive Assessment; CI, confidence interval.

**Supplementary Table S5 Changes in CBF of cognition-related brain regions at Baseline and Post-Intervention.**

| Brain Region | Health Education control Group (n = 20) | | | | Tai Chi Group (n = 20) | | | | Cohen's *d* | Between-group *P*-value |
| --- | --- | --- | --- | --- | --- | --- | --- | --- | --- | --- |
|  | Pre | Post | d | within-group *P*-value | Pre | Post | d | within-group *P*-value |  |  |
| Left Middle Frontal Gyrus | 52.32 ± 4.99 | 47.86 ± 5.64 | -4.46 ± 4.29 | **< 0.001^**^** | 51.01 ± 4.16 | 57.95 ± 4.93 | 6.93 ± 5.90 | **< 0.001^**^** | 2.21 | **< 0.001^##^** |
| Right Middle Frontal Gyrus | 51.78 ± 4.89 | 47.74 ± 5.46 | -4.04 ± 2.78 | **< 0.001^**^** | 50.88 ± 3.58 | 58.75 ± 6.22 | 7.87 ± 6.75 | **< 0.001^**^** | 2.31 | **< 0.001^##^** |
| Left Anterior Cingulate Cortex | 51.81 ± 3.46 | 48.44 ± 3.57 | -3.37 ± 5.65 | **0.015^*^** | 50.92 ± 4.75 | 60.99 ± 6.45 | 10.07 ± 5.95 | **< 0.001^**^** | 2.32 | **< 0.001^##^** |
| Right Anterior Cingulate Cortex | 51.60 ± 3.60 | 48.64 ± 3.40 | -2.96 ± 5.15 | **0.019^*^** | 50.26 ± 4.93 | 60.31 ± 6.28 | 10.05 ± 4.83 | **< 0.001^**^** | 2.61 | **< 0.001^##^** |
| Left Hippocampus | 49.34 ± 3.79 | 46.82 ± 3.81 | -2.53 ± 4.43 | **0.020^*^** | 48.80 ± 3.74 | 52.85 ± 4.07 | 4.05 ± 3.75 | **< 0.001^**^** | 1.60 | **< 0.001^##^** |
| Right Hippocampus | 49.02 ± 4.47 | 46.47 ± 4.24 | -2.56 ± 4.77 | **0.027^*^** | 49.00 ± 3.71 | 54.47 ± 4.36 | 5.47 ± 4.32 | **< 0.001^**^** | 1.77 | **< 0.001^##^** |
| Left Parahippocampal Gyrus | 50.61 ± 3.18 | 46.46 ± 3.40 | -4.15 ± 4.28 | **< 0.001^**^** | 49.50 ± 3.04 | 60.12 ± 5.60 | 10.62 ± 4.21 | **< 0.001^**^** | 3.48 | **< 0.001^##^** |
| Right Parahippocampal Gyrus | 51.48 ± 4.33 | 47.45 ± 2.98 | -4.03 ± 5.04 | **0.002^**^** | 49.58 ± 3.53 | 60.91 ± 6.79 | 11.33 ± 5.45 | **< 0.001^**^** | 2.93 | **< 0.001^##^** |
| Global mean CBF | 51.58 ± 3.74 | 46.58 ± 4.23 | -5.00 ± 5.24 | **< 0.001^**^** | 49.84 ± 3.87 | 60.13 ± 4.36 | 10.29 ± 2.87 | **< 0.001^**^** | 3.62 | **< 0.001^##^** |

Note: All CBF values are presented as mean ± SD, with the unit of mL·100 g^-1^·min^-1^. d = change score (post-intervention minus baseline). Within-group comparisons: paired *t*-tests, ^*^*P* < 0.05, ^**^*P* < 0.01 vs. baseline. Between-group comparisons: independent samples *t*-tests, ^##^*P* < 0.01 vs. the health education control group. Cohen's *d*: 0.2 = small, 0.5 = medium, 0.8 = large effect.

Abbreviation: CBF, cerebral blood flow; SD, standard deviation.

**Supplementary Table S6** Changes in Circulating Vasomotor Factors and Serum AD-related Biomarkers at Baseline and Post-Intervention.

| Outcome | Health Education control Group (n = 20) | | | | Tai Chi Group (n = 20) | | | | Cohen's *d* | Between-group *P*-value |
| --- | --- | --- | --- | --- | --- | --- | --- | --- | --- | --- |
|  | Pre | Post | d | within-group *P*-value | Pre | Post | d | within-group *P*-value |  |  |
| NO (μmol/L) | 49.74 ± 6.37 | 49.72 ± 7.84 | -0.02 ± 10.85 | 0.994 | 49.13 ± 3.71 | 59.52 ± 5.51 | 10.39 ± 5.50 | **< 0.001^**^** | 1.21 | **0.001^##^** |
| ET-1 (pg/mL) | 159.91 ± 28.93 | 110.34 ± 33.28 | -49.57 ± 43.96 | **< 0.001^**^** | 171.73 ± 34.00 | 93.13 ± 34.59 | -78.61 ± 42.11 | **< 0.001^**^** | 0.68 | **0.039^#^** |
| PGI_2_ (pg/mL) | 79.95 ± 14.46 | 189.77 ± 28.13 | 109.82 ± 26.58 | **< 0.001^**^** | 85.87 ± 17.00 | 178.29 ± 36.96 | 92.42 ± 36.07 | **< 0.001^**^** | 0.55 | 0.091 |
| TXA_2_ (pg/mL) | 251.05 ± 42.53 | 183.01 ± 43.06 | -68.04 ± 65.65 | **< 0.001^**^** | 236.22 ± 37.21 | 169.93 ± 44.98 | -66.30 ± 51.05 | **< 0.001^**^** | 0.03 | 0.926 |
| ET-1/NO | 3.28 ± 0.82 | 2.32 ± 0.92 | -0.97 ± 1.19 | **0.002^**^** | 3.50 ± 0.67 | 1.56 ± 0.54 | -1.94 ± 0.74 | **< 0.001^**^** | 0.98 | **0.004^##^** |
| TXA_2_/PGI_2_ | 3.25 ± 0.81 | 1.00 ± 0.34 | -2.25 ± 0.78 | **< 0.001^**^** | 2.85 ± 0.73 | 1.01 ± 0.37 | -1.85 ± 0.63 | **< 0.001^**^** | 0.56 | 0.084 |
| T-tau (pg/mL) | 644.52 ± 125.65 | 670.75 ± 118.32 | 26.23 ± 154.60 | 0.457 | 685.19 ± 123.95 | 455.97 ± 90.88 | -229.22 ± 137.55 | **< 0.001^**^** | 1.75 | **< 0.001^##^** |
| P-tau (pg/mL) | 581.06 ± 94.67 | 536.72 ± 101.11 | -44.34 ± 96.98 | 0.055 | 599.91 ± 89.09 | 419.89 ± 91.90 | -180.01 ± 108.90 | **< 0.001^**^** | 1.32 | **< 0.001^##^** |
| Aβ_1_-42 (pg/mL) | 707.61 ± 96.85 | 683.72 ± 110.80 | -23.90 ± 107.13 | 0.331 | 678.94 ± 127.94 | 485.63 ± 104.38 | -193.30 ± 157.78 | **< 0.001^**^** | 1.26 | **< 0.001^##^** |

Note: Data are presented as mean ± SD. d = change score (post-intervention minus baseline). Within-group comparisons: paired *t*-tests, ^**^*P* < 0.01 vs. baseline. Between-group comparisons: independent samples *t*-tests, ^#^*P* < 0.05, ^##^*P* < 0.01 vs. the health education control group. Cohen's *d*: 0.2 = small, 0.5 = medium, 0.8 = large effect. Abbreviations: NO, nitric oxide; ET-1, endothelin-1; PGI_2_, prostacyclin; TXA_2_, thromboxane A_2_; T-tau, total tau; P-tau, phosphorylated tau; Aβ_1_-42, amyloid-beta_1_-42; SD, standard deviation.

**Supplementary Figures**

**Supplementary Figure S1** Individual-level distributions of changes from baseline to post-intervention in MoCA total score and three domain-specific scores.

A. Change in MoCA total score; B. Change in delayed recall score; C. Change in visuospatial/executive score; D. Change in attention score.

Note: Changes were calculated as post-intervention scores minus baseline scores. Each dot represents an individual participant. Box plots indicate the median and interquartile range (IQR), with whiskers representing 1.5×IQR. Error bars = SD. Between-group comparisons: Mann-Whitney U test. ^***^*P* < 0.001 vs. the health education control group. Abbreviation: MoCA, Montreal Cognitive Assessment; SD, standard deviation.

**Supplementary Figure S2** Individual-level distributions of changes from baseline to post-intervention in global mean CBF and CBF of cognition-related brain regions.

A. Change in global mean CBF; B. Change in CBF of left middle frontal gyrus; C. Change in CBF of right middle frontal gyrus; D. Change in CBF of left anterior cingulate cortex; E. Change in CBF of right anterior cingulate cortex; F. Change in CBF of left hippocampus; G. Change in CBF of right hippocampus; H. Change in CBF of left parahippocampal gyrus; I. Change in CBF of right parahippocampal gyrus.

Note: Changes were calculated as post-intervention values minus baseline values. Each dot represents an individual participant. Box plots indicate the median and interquartile range (IQR), with whiskers representing 1.5×IQR. Error bars = SD. Between-group differences: independent samples *t*-tests, ^***^*P* < 0.001 vs. the health education control group. Abbreviations: CBF, cerebral blood flow; SD, standard deviation.

**Supplementary Figure S3** Individual-level distributions of changes from baseline to post-intervention in circulating vasomotor and serum AD-related biomarkers.

A. Change in plasma NO; B. Change in plasma ET-1; C. Change in ET-1/NO ratio; D. Change in serum T-tau; E. Change in serum P-tau; F. Change in serum Aβ_1_-42.

Note: Changes were calculated as post-intervention values minus baseline values. Each dot represents an individual participant. Box plots indicate the median and interquartile range (IQR), with whiskers representing 1.5×IQR. Error bars = SD. Between-group differences: independent samples *t*-tests, ^***^*P* < 0.001 vs. the health education control group. Abbreviations: NO, nitric oxide; ET-1, endothelin-1; T-tau, total tau; P-tau, phosphorylated tau; Aβ_1_-42, amyloid-beta 1-42; SD, standard deviation.
